# Randomized phase II trial of weekly ixabepilone ± biweekly bevacizumab for platinum-resistant or refractory ovarian/fallopian tube/primary peritoneal cancer (NCT03093155): Updated survival and subgroup analyses

**DOI:** 10.1038/s44276-024-00067-5

**Published:** 2024-06-20

**Authors:** Dana M. Roque, Eric R. Siegel, Natalia Buza, Stefania Bellone, Gloria S. Huang, Gary Altwerger, Vaagn Andikyan, Mitchell Clark, Masoud Azodi, Peter E. Schwartz, Gautam G. Rao, Elena Ratner, Alessandro D. Santin

**Affiliations:** 1 Division of Gynecologic Oncology, Marlene and Stewart Greenebaum Comprehensive Cancer Center, University of Maryland School of Medicine, Baltimore, MD USA; 2 Department of Biostatistics, University of Arkansas for Medical Sciences, Little Rock, AR USA; 3 Department of Pathology, Smilow Comprehensive Cancer Center, Yale School of Medicine, New Haven, CT USA; 4 Division of Gynecologic Oncology, Smilow Comprehensive Cancer Center, Yale School of Medicine, New Haven, CT USA

## Abstract

**Background:**

Ixabepilone may retain activity in paclitaxel-resistant disease. We previously reported improved response rates (ORR), progression-free (PFS), and overall survival (OS) conferred by ixabepilone+bevacizumab (IXA + BEV) compared to monotherapy (IXA) in heavily pre-treated ovarian cancers. We now describe a mature data set. Subset analyses were performed in patients with different taxane sensitivities and dose modifications.

**Methods:**

Patients previously treated with paclitaxel were stratified by prior BEV and randomized to receive IXA 20 mg/m^2^ days 1,8,15 ± BEV 10 mg/kg days 1,15 of a 28-day cycle in a multi-site prospective randomized phase 2 trial.

**Results:**

Thirty-seven patients were randomized to IXA and 39 patients to IXA + BEV. At the final data cutoff (05/27/2023), ORR was higher in the IXA + BEV arm (38.4% vs. 8.1%, *p* = 0.003). Dose reductions were necessary in most participants but did not diminish PFS/OS benefits. Most patients were paclitaxel-refractory/-resistant (51%, *n* = 19/37;67%, *n* = 26/39); the remainder were taxane-sensitive. The addition of BEV to IXA conferred benefit in PFS (5.5 vs. 2.2 mo; HR 0.31, 90%CI 0.20–0.49, *p* < 0.001) and OS (10.3 vs. 6.0 mo; HR 0.56, 90%CI 0.38–0.84, *p* = 0.02) that persisted after adjusting for prior taxane response.

**Conclusions:**

IXA + BEV has activity in heavily pre-treated ovarian cancers and offers significant improvement in ORR and PFS/OS compared to IXA, despite prior taxane response and dose reductions.

**Clinical Trial Registration:**

NCT03093155

## Introduction

Platinum and taxane combination chemotherapy represents the backbone of ovarian cancer treatment in the upfront setting [1, 2]. The combination of weekly paclitaxel with bevacizumab constitutes one of the most active regimens for platinum-resistant disease in patients treated with two or fewer prior lines of therapy [3, 4]. Unfortunately, heavily pre-treated patients continue to suffer from a lack of effective treatment options. After 5 lines, response rates are as low as 10–16% [5] and median overall survival after 3 lines may be as low as 5–9 months[6].

Proposed mechanisms of taxane resistance include enhanced drug efflux from the cell, rapid drug metabolism, dysregulation of microtubule-stabilizing proteins, altered binding affinity, upregulation of mediators of cell cycle progression, blunted spindle cell assembly checkpoint signals[7], and alterations in signal transduction pathways that affect autophagy, senescence, and inflammation [8]. Though structurally distinct from taxanes, epothilones, such as ixabepilone (Ixempra® R-Pharm US, NJ), also hyperstabilize microtubules and may continue to exert effects in taxane-treated patients by overcoming these common resistance mechanisms [9, 10].

We conducted a phase II prospective multi-site comparison of ixabepilone with bevacizumab (IXA + BEV) versus ixabepilone monotherapy (IXA). With an initial data analysis date of 11/2020, the previous publication [11] showed IXA + BEV to be an effective combination for heavily pre-treated (median of 4 prior lines) platinum-resistant and taxane-treated ovarian cancer patients. We found that the benefits of combination therapy on progression-free (PFS) and overall survival (OS) were not diminished by prior receipt of bevacizumab. IXA dose reductions were required by approximately 60% of participants in each arm most commonly due to peripheral neuropathy, neutropenia, and fatigue. The National Comprehensive Cancer Network (NCCN®) subsequently endorsed this combination as a category 2B treatment option for recurrent, platinum-resistant ovarian disease [2].

In the current manuscript, we present the final survival analyses of the mature clinical trial data and further characterize the performance of the regimen in patients previously treated with weekly paclitaxel and among those requiring dose reduction.

## Methods

### Study design and conduct

**Fig. 1:**
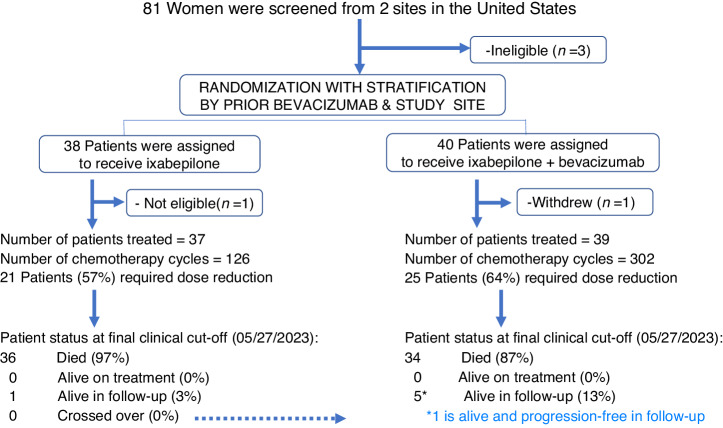
CONSORT (Consolidated Standards of Reporting Trials) diagram.

This was an investigator-initiated phase II randomized open-label trial (NCT03093155) conducted at Smilow Cancer Hospital at Yale University and the Greenebaum Comprehensive Cancer Center at the University of Maryland. As previously described [11], study participants were stratified by study site and previous receipt of BEV with a 1:1 allocation using a dynamic randomization procedure to minimize stratification-factor imbalance between arms. IXA monotherapy at 20 mg/m^2^ intravenously on days 1, 8, and 15 of a 28-day cycle was administered alone or with BEV 10 mg/kg intravenously days 1 and 15 administered until disease progression, death, or prohibitive toxicity (Fig. 1). In the original publication, the CONSORT diagram listed the number of infusions as ‘cycles;’ this has been updated in the present work to reflect true cycles. There were no significant amendments made during the conduct of the trial, though enrollment was briefly suspended during the COVID-19 pandemic. Power calculations assumed a median PFS for IXA monotherapy to be 5 months, given observations from GOG 126 M [12]. We required 80% power at 5% $\alpha$ to detect a 2-fold increase in PFS via a one-sided log-rank test while allowing for a single interim analysis for efficacy and futility. Calculations conducted in East v 6.4 (Cytel, Inc, Cambridge, MA) using the null variance estimator along with the O’Brien-Fleming spending functions for both alpha and beta required 28 PFS events in the interim and 56 PFS events in the final analysis. The first participant enrolled in March 2017. Because the COVID-19 pandemic slowed recruitment, we terminated enrollment after 78 participants and the occurrence of 61 PFS events. The present analyses represent a data cut-off of 05/27/2023. The full protocol is provided in the Supplementary Appendix. Similar to previously published definitions by others [13], patients were considered *taxane-resistant* if they demonstrated disease progression within 6 months of paclitaxel/docetaxel administration. Patients were considered *taxane-refractory* if they progressed while receiving a taxane or demonstrated persistence of disease on the end-of-treatment assessment that prompted the initiation of a new line of therapy. By default, the remaining patients were considered *taxane-sensitive*. This investigation was conducted in accordance with the Declaration of Helsinki and approved by the local Institutional Review Board. All patients provided written informed consent.

### Eligibility

Participants must have received prior treatment with $\ge$3 cycles paclitaxel, either 3-weekly or weekly. There was no limit on prior lines including BEV therapy. All participants were $\ge$18 years old and had platinum-resistant (i.e., platinum-free interval <6 months) or refractory (i.e., disease progression during or $\le$4 weeks after the last dose of platinum) histologically confirmed epithelial (non-mucinous) ovarian, fallopian tube, or primary peritoneal carcinoma. All participants had measurable disease per RECIST (Response Evaluation Criteria in Solid Tumors) v1.1 [14]. Participants had to exhibit a performance status of 0–2 [15]. Any prior debulking status was permitted.

### Study drugs

IXA was provided by R-Pharm US LLC, Princeton, NJ. BEV was supplied commercially. Biosimilars were not permitted. Two 20% dose reductions were allowed for ixabepilone to 16 mg/m^2^ and then 12 mg/m^2^

### Endpoints

Computed tomography was performed every 2 cycles. The primary endpoint was PFS, defined as the time from randomization to progression or death. Secondary endpoints were OS, defined as the time from randomization to death from any cause, and safety as defined by Common Terminology Criteria for Adverse Events (CTCAE) v.4 [16]. Best response was based on RECIST v1.1. OR consisted of complete response (CR) or partial response (PR), and it did not have a durability requirement. Durable Disease Control (DDC) was defined as CR, PR, or stable disease (SD) $\ge$6 months from the date of best response.

### Statistical analyses

PFS and OS were analyzed using the Kaplan-Meier method with one-sided log-rank tests and Cox regression. Multi-variate analyses were performed with paclitaxel response as a co-variate (sensitive, resistant, refractory). Fisher’s exact tests were used to compare differences in best response between arms across subgroups. Individual patient responses were illustrated as a swimmer’s plot. The datasets generated and analyzed during the current study are available from the corresponding author on request.

## Results

### Treatment and disease characteristics

Eighty-one participants were screened, and 78 were randomized (Fig. 1). One withdrew consent and one was found ineligible, leaving 76 evaluable for efficacy. Patient and disease characteristics showed no evidence of imbalance [11]. Notably, 49% of participants had received >3 prior lines of chemotherapy and 18% had platinum-refractory disease, with no statistical differences between the arms (*p* = 0.82 and *p* = 0.14, respectively). Within the IXA arm (*N* = 37), 9 (24%) of patients were taxane-resistant, 10 (27%) were taxane-refractory; within the IXA + BEV arm (*N* = 39), 13 (33%) were taxane-resistant, 13 (33%) were taxane-refractory (*p* = 0.44) (Table 1). Both arms contained patients previously treated with weekly paclitaxel, either in combination with carboplatin or in the platinum-resistant setting as part of an AURELIA [4] regimen: 35% (*N* = 13; IXA) and 26% (*N* = 10, IXA + BEV). (A correction has been submitted to the original manuscript due to an error in Table 1. We previously reported 27% (*N* = 10) of patients in the IXA arm and 23% (*N* = 9) in the IXA+BEV arm had been treated with weekly paclitaxel, either with carboplatin or as an AURELIA regimen with or without bevacizumab. Among these patients, we described 1 patient in the IXA arm and 4 patients in the IXA+BEV arm who had received weekly paclitaxel with bevacizumab and 1 patient in the IXA arm and 2 in the IXA+ BEV arm who received weekly paclitaxel monotherapy.) One additional patient in the IXA + BEV arm also received weekly nab-paclitaxel but was not included for purity of the analysis. Among all patients previously treated with weekly paclitaxel, two patients in each arm (5.4% and 5.1%, respectively) had received weekly paclitaxel with bevacizumab and two patients in each arm had received weekly paclitaxel monotherapy. Of note, one patient received both weekly paclitaxel followed 16 months later by weekly paclitaxel with bevacizumab and is therefore counted towards both totals.

**Table 1:** Patient characteristics.

	IXA (*n* = 37)	IXA+BEV (*n* = 39)
**Age in years**, median (range)	67 (50–88)	67 (40–78)
**Race, % (*N*)**		
White	73% (27)	80% (31)
Black	22% (8)	10% (4)
Other	5% (2)	10%(4)
**Ethnicity, % (*N*)**		
Hispanic	8% (3)	3% (1)
Non-Hispanic	92% (34)	95% (37)
Unknown	0	3% (1)
**Histology, % (*N*)**		
Serous	78% (29)	87% (34)
Carcinosarcoma	6% (2)	3% (1)
Other	16% (6)	10% (4)
**ECOG Performance Status, % (*N*)**		
0–1	84% (31)	92% (36)
2	16% (6)	8 % (3)
**Prior Lines of Chemotherapy, % (*N*)**		
≤3	49% (18)	54% (21)
>3	51% (19)	46% (18)
**Prior PARP Inhibitor**		
Yes	38% (14)	26% (10)
No	62% (23)	74% (29)
**Prior Bevacizumab, % (*N*)**		
Yes	57% (21)	54% (21)
No	43% (16)	46% (18)
**Prior Weekly Paclitaxel (for first line treatment or treatment of recurrence), % (*N*)**		
Yes	35% (13)	26% (10)
No	65% (24)	74% (29)
**Prior Receipt of an AURELIA Regimen, % (*N*)**		
- With Bevacizumab		
Weekly paclitaxel	29% (2)	33% (2)
Pegylated liposomal doxorubicin	29% (2)	66% (4)
Topotecan	14% (1)	0
> 1 AURELIA regimen	29% (2)	0
- Without Bevacizumab		
Weekly paclitaxel	17% (2)	11% (2)
Pegylated liposomal doxorubicin	58% (7)	67% (12)
Topotecan	8% (1)	17% (3)
>1 AURELIA regimen	17% (2)	5% (1)
**Platinum Refractory/Resistant Disease, % (N)**		
Refractory	11% (4)	26% (10)
Resistant	89% (33)	74% (29)
**Taxane Refractory/Resistant Disease, % (N)**		
Refractory	27% (10)	33% (13)
Resistant	24% (9)	33% (13)
Sensitive	49% (18)	33% (13)

*ECOG* Eastern Cooperative Oncology Group

### Primary endpoint: PFS

At the data cutoff (05/27/2023), 75 PFS events and 70 deaths had occurred among 76 participants during 94.37 person-years of observation. Median PFS was 5.5 versus 2.2 months, HR 0.31, 90%CI 0.20–0.49, *p* < 0.001); this is unchanged from the previous estimates.

### Secondary endpoints: OS, OR rate (ORR), safety

**Fig. 2:**
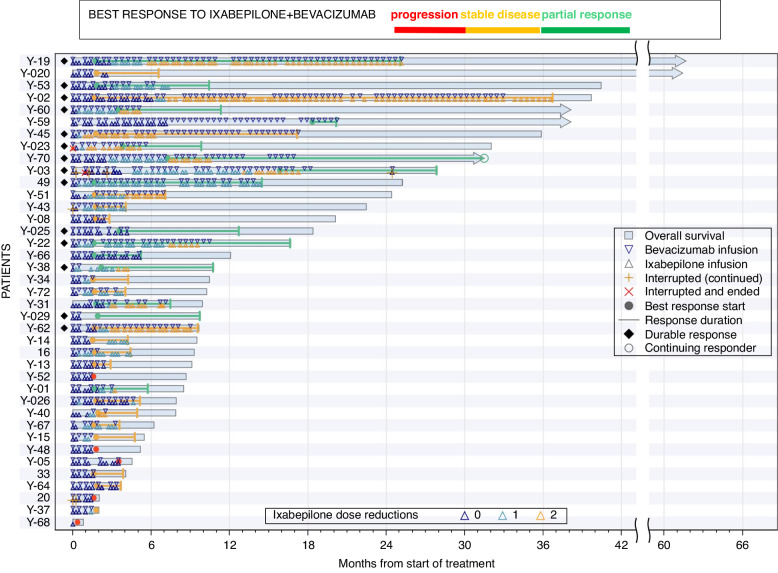
Swimmer’s plot of responses among 39 patients who received ixabepilone and bevacizumab.

Since the original report, there has been one additional response. ORR increased and remained higher in the IXA + BEV arm (38.4% vs. 8.1%, *p* = 0.003). Median OS was 10.3 versus 6.0 months (HR 0.56, 90%CI 0.38–0.84, *p* = 0.02); this is unchanged from the previous estimates. There were no complete responses, but in the combination arm, 14 patients achieved a durable response (stable disease or partial response > 6 months) (Fig. 2).

**Fig. 3:**
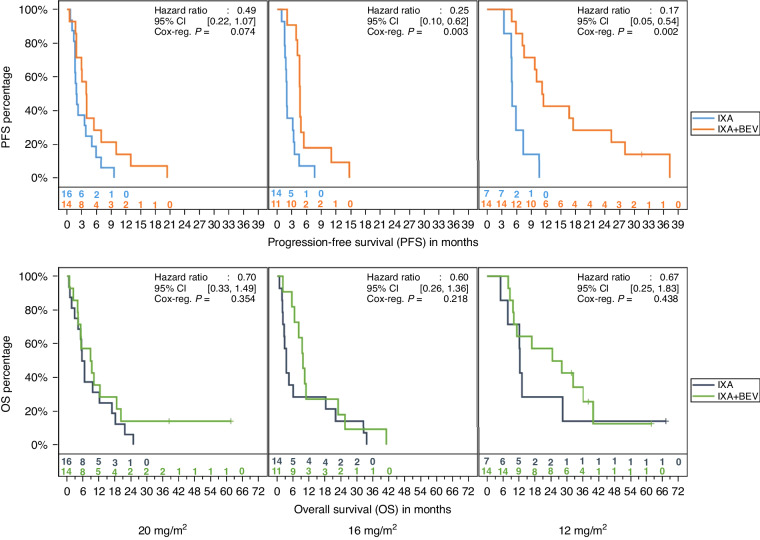
Progression-free (top) and overall (bottom) survival among patients in the setting of ixabepilone dose reductions (left, none; middle, one dose reduction, right, two dose reductions).

No new safety signals emerged since the original report. Among all participants, there were 30 patients who tolerated the initially prescribed dose, 25 patients who received at least one cycle at one dose reduction (16 mg/m^2^) and 21 who received at least one cycle at a second dose reduction (12 mg/m^2^). PFS benefit persisted in the combination arm despite dose reduction (HR 0.49, 95%CI 0.22–1.07, *p* = 0.074 [full dose]; HR 0.25, 95%CI 0.10–0.62, *p* = 0.003 [one dose reduction], HR 0.17, 95%CI 0.05–0.54, *p* = 0.002 [two dose reductions]) (Fig. 3-upper). OS was not affected (HR 0.7, 95%CI 0.33–1.49, *p* = 0.354 [full dose]; HR 0.6, 95%CI 0.26–1.36, *p* = 0.218 [one dose reduction]; HR 0.67, 95% CI 0.25–1.83, *p* = 0.438) [two dose reductions] (Fig. 3-lower).

### Subgroup analyses: prior taxane response and prior exposure to weekly paclitaxel

Among both arms, most patients were paclitaxel-refractory/-resistant (51% [*N* = 19/37] on IXA and 67% [*N* = 26/39] on IXA + BEV. The addition of BEV to IXA conferred benefit in PFS (HR 0.31, 90%CI 0.20–0.48, 2-tailed Wald *p* < 0.001) and OS (HR 0.59, 90%CI 0.39–0.88, 2-tailed Wald *p* = 0.03) even in a multivariate analysis with prior taxane response as a covariate. Among 10 taxane-refractory patients in the IXA arm, the best responses included SD in 40% (*N* = 4) and progressive disease (PD) in 50%(*N* = 5); one patient was not assessed due to rapid death. Among 13 taxane-refractory patients in the IXA + BEV arm, the best responses included PR in 30.8% (*N* = 4), SD in 30.8% (*N* = 4) SD, and PD in 38.5% (*N* = 5). Although the likelihood of increased PR with BEV did not attain statistical significance within any one prior-taxane subgroup (lowest *p* = 0.06), a Cochran-Mantel-Haenszel analysis with strata defined by prior taxane response yielded a common odds ratio of 0.71 (95%CI: 1.77–28.8; *p* = 0.003) favoring PR with IXA + BEV.

**Fig. 4:**
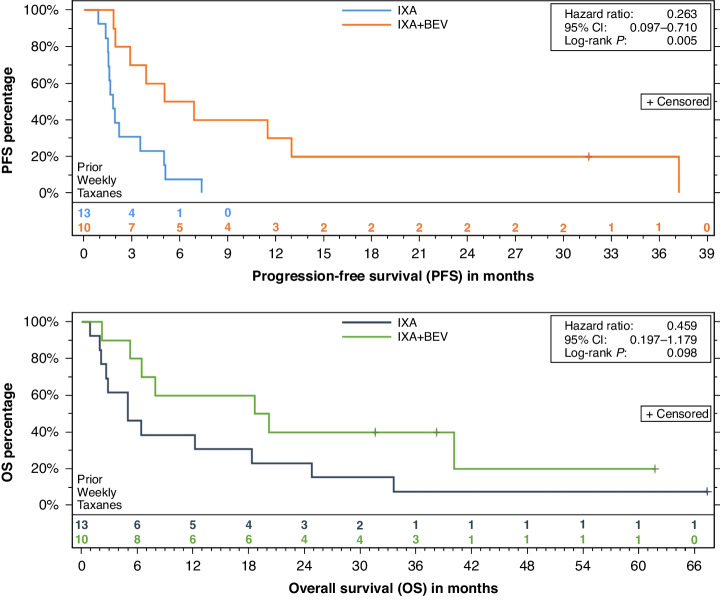
Progression-free (top) and overall (bottom) survival among patients in 23 patients who received prior weekly paclitaxel.

Among all 23 patients with prior weekly paclitaxel exposure (either in conjunction with carboplatin or as an AURELIA regimen in the recurrent setting), PFS was 6.0 (95%CI 2.9–13.0, IXA + BEV) versus 1.8 (95%CI 1.5–3.5, IXA) months (log-rank *p* = 0.005) (Fig. 4-upper; HR 0.26, 95%CI 0.10–0.71). OS estimates were 19.4 (95%CI 6.4 to not reached; IXA + BEV) versus 5.0 (95%CI 2.6–18.3, IXA) months (log-rank *p* = 0.10) (Fig. 4-lower; HR 0.46, 95%CI 0.18–1.18). In the subset of these patients who had received an AURELIA regimen (i.e., weekly paclitaxel with or without bevacizumab), PFS estimates were 2.9 (95%CI 2.0–6.9, IXA + BEV) and 1.7 (95%CI 0.9–2.2, IXA) months (*p* = 0.07). Following receipt of an AURELIA regimen of weekly paclitaxel with or without BEV, OS estimates were 20.2 (95%CI 2.2-not reached, IXA + BEV) and 4.5 (95%CI 0.9 to 18.3, IXA) months (*p *= 0.17). Both patients treated with IXA + BEV following prior weekly paclitaxel and bevacizumab achieved the best response of SD, as did both patients treated with IXA + BEV following prior weekly paclitaxel monotherapy. One of two patients treated with IXA following prior weekly paclitaxel and bevacizumab achieved the best response of SD; one patient treated with IXA following prior weekly paclitaxel monotherapy experienced PD and one was not assessed due to death. The distribution of responses for all patients who received prior weekly paclitaxel is provided in Table 2. Among these 23 patients, there were 3 PRs in the combination arm but no responses in the mono therapy arm (*p* = 0.07). DDC was achieved in 4 patients in the combination arm but in none among the monotherapy arm (*p* = 0.02).

**Table 2:** Best response among patients treated with weekly paclitaxel.

Arm	Best Response	Median Follow-Up	Expired?	Prior weekly Paclitaxel + Carboplatin	Prior weekly Paclitaxel + Bevacizumab	Prior Weekly Paclitaxel
IXA	Not Assessed	0.89	Yes			Yes
IXA	Progressive Disease	2.63	Yes		Yes	
IXA	Progressive Disease	2.83	Yes	Yes		
IXA	Progressive Disease	5	Yes	Yes		
IXA	Progressive Disease	18.31	Yes			Yes
IXA	Progressive Disease	24.79	Yes	Yes		
IXA	Stable Disease	1.94	Yes	Yes		
IXA	Stable Disease	2.1	Yes	Yes		
IXA	Stable Disease	5	Yes	Yes		
IXA	Stable Disease	6.41	Yes		Yes	
IXA	Stable Disease	12.2	Yes	Yes		
IXA	Stable Disease	33.6	Yes	Yes		
IXA	Stable Disease	67.36	No	Yes		
IXA+BEV	Progressive Disease	5.23	Yes	Yes		
IXA+BEV	Stable Disease	2.17	Yes		Yes	
IXA+BEV	Stable Disease	6.44	Yes	Yes		
IXA+BEV	Stable Disease	7.92	Yes	Yes		
IXA+BEV	Stable Disease	20.15	Yes			Yes
IXA+BEV	Stable Disease	40.11	Yes	Yes		
IXA+BEV	Stable Disease	61.71	No		Yes	Yes
IXA+BEV	Partial Response	18.61	Yes	Yes		
IXA+BEV	Partial Response	31.59	No	Yes		
IXA+BEV	Partial Response	38.24	No	Yes		

## Discussion

We previously reported improved ORR, PFS, and OS conferred by weekly ixabepilone with biweekly bevacizumab (IXA + BEV) compared to monotherapy (IXA) in heavily pre-treated ovarian cancers. We suggested that prior BEV should not preclude re-treatment with the combination of IXA + BEV and highlighted pre-clinical observations that may explain the apparent synergy between these two agents [11]. The present report also illustrates encouraging activity of IXA + BEV in patients despite prior exposure to weekly paclitaxel and in the setting of dose reductions to further inform clinicians following endorsement of the combination by the NCCN (category 2B) [2].

The mature data reveal an impressive ORR of the combination arm (38.4% vs. 8.1%, *p* = 0.003), with durable responses in 14 patients and respectable median PFS of 5.5 months and OS of 10 months. Unsurprisingly, in a heavily pre-treated population, dose reductions were common, but these ancillary analyses suggest this did not damper response and allowed patients to continue therapy. Our data  support consideration for a starting dose of 16 mg/m^2^ .

While favorable responses to weekly paclitaxel with [17] or without bevacizumab [18, 19] after three-weekly paclitaxel are well-described, data quantifying the response to repeated treatment with weekly paclitaxel remain limited. Gunderson et al (2017) reported no attenuation of benefit for weekly paclitaxel despite prior exposure to weekly administration during primary therapy among 20 patients; compared to 79 patients previously exposed to three-weekly administration, the clinical benefit rate was actually improved (69% versus 36%, *p* = 0.05) [20]. In another small series of 26 patients [21], re-challenge with weekly paclitaxel after prior weekly paclitaxel produced a radiologic response rate of 34.6% with a median PFS of 3.7 months (95%CI 2.3–6.7) and OS of 18.1 (95%CI 9.6–28.6). Three patients received weekly paclitaxel for a 3rd time and 2/3 achieved stable disease. It is therefore not unreasonable to expect a subset of patients to benefit from dose-dense treatment with another microtubule-stabilizing agent such as ixabepilone following prior weekly paclitaxel. Ixabepilone may overcome paclitaxel resistance in several ways. Firstly, epothilones tend not to be substrates of drug exportation pumps that shuttle paclitaxel from the cell. Secondly, epothilones also appear to retain affinity for the microtubule despite upregulation of class III β-tubulin over the constitutively expressed class I β-tubulin, which reduces paclitaxel binding by altering the sterics of the pocket [9, 10]. Exploratory analyses employing whole exome sequencing among patients with a response to weekly paclitaxel for > 12 months suggested the importance of genes related to angiogenesis (VEGF, MMP9), tubulin superfamily (TSC2), apoptosis (BCL2L1, BAD) and interleukin pathways (CXCR1, CXCR2, IL1A, IL1B) [22]. Studies investigating predictors of response to weekly paclitaxel are surprisingly scant, and are also lacking for epothilones, and should be the subject of future investigations.

In summary, the regimen of weekly ixabepilone and biweekly bevacizumab achieves high response rates and respectable PFS and OS in heavily pre-treated ovarian cancer patients. Response rates appear to be higher than those reported in this setting for chemotherapy alone, as well as for non-taxane chemotherapies combined with bevacizumab. Use of the combination does not appear to be precluded by exposure to prior bevacizumab or previous treatment with weekly paclitaxel. Biomarkers to predict sustained response to treatment with weekly ixabepilone and bevacizumab are needed.

## Supplementary information

Supplementary information

## Data Availability

The datasets generated and/or analyzed during the current study are available from the corresponding author on reasonable request.
