# Supplementary material for: Randomized phase II trial of weekly ixabepilone ± biweekly bevacizumab for platinum-resistant or refractory ovarian/fallopian tube/primary peritoneal cancer (NCT03093155): Updated survival and subgroup analyses
Source: BJC Rep. 2024 Jun 20;2:43. doi: 10.1038/s44276-024-00067-5 (PMC11523995; doi:10.1038/s44276-024-00067-5)
Supplement: Supplementary file 1 — Supplementary information [file 44276_2024_67_MOESM1_ESM.pdf]

A randomized phase II evaluation of weekly ixabepilone with or without biweekly bevacizumab in recurrent or persistent platinum-resistant/refractory epithelial ovarian, fallopian tube, or primary peritoneal cancers

STUDY CONTACTS

STUDY PI:
Alessandro D. Santin MD
Yale University School of Medicine
Division of Gynecologic Oncology
Department of Obstetrics, Gynecology & Reproductive Sciences
333 Cedar Street, LSOG Bld. Room 305, PO Box 208063
New Haven, CT, 06520-8063
Phone: 203-737-4450
Fax: 203-737-4339
E-mail: alessandro.santin@yale.edu

Co-PI:
Dana Marie Roque, MD
University of Maryland School of Medicine
Division of Gynecologic Oncology
Department of Obstetrics, Gynecology & Reproductive Sciences
22 South Greene Street S3AX31
Baltimore, MD 21201
Phone: 410-328-2076
Fax: 410-328-8389
E-mail: droque@fpi.umaryland.edu

GYNECOLOGIC PATHOLOGIST:
Natalia Buza, MD
Yale University
Department of Pathology
310 Cedar St. LH 222
New Haven, CT 06510
Phone: 203-785-5439
Fax: 203-737-2470
E-mail: natalia.buza@yale.edu

STATISTICIAN:
Eric R. Siegel MS
Department of Biostatistics
University of Arkansas for Medical Sciences,
4301 W. Markham, Little Rock,
Little Rock, AR, 72205-7199.
Phone: 501-526-6717
Fax: 501-526-6717

E-mail: SiegelEricR@uams.edu

MULTI-SITE /REMOTE MONITORING COORDINATOR:
Lisa Baker RN, BSN, OCN
Clinical Research Coordinator
Gyn-Oncology
Yale University School of Medicine
Phone: 203-785-6398
Fax 203-785-7480
E-mail: lisa.baker@yale.edu

CLINICAL RESEARCH COORDINATORS:
Lisa Baker RN, BSN, OCN and Marthanne Luther RN, MPH
Gyn-Oncology
Yale University School of Medicine
Phone: 203-785-6398; 203-737-2781
Fax 203-785-7480
E-mail: lisa.baker@yale.edu; martha.luther@yale.edu

REGULATORY COORDINATOR:
Kay Debski, MPH
Gyn-Oncology
Yale University School of Medicine
Phone: (203) 737-5653
Fax: 203-785-7480
E-mail: kay.debski@yale.edu

SPECIMEN PROCUREMENT COORDINATOR/ MULTI-SITE DATA MANAGER:
Michele Montagna, MS
Yale University
FMB 340, New Haven, CT 06520
Telephone (203)737-7660 Fax (203) 785-7480
E-mail: michele.montagna@yale.edu

MULTI-SITE ADMINISTRATIVE ASSISTANT:
Lisa Patriub
Gyn-Oncology
Yale School of Medicine
Phone: 203-737-2280
Fax: 203-737-4339
E-mail: lisa.patriub@yale.edu

Recurrent or Persistent Platinum-Resistant/Refractory Epithelial (non-mucinous) Ovarian, Fallopian tube, or Primary Peritoneal Cancer with Measureable Disease

SCHEMA

Tumor Specimen Available for Immunohistochemistry (IHC)

Stratification by Prior Receipt of Bevacizumab

YES	NO

RANDOMIZATION

Ixabepilone 20 mg/m^2<br>days 1, 8, 15<br>Q 28 days<br>(A: control arm)	Ixabepilone 20 mg/m^2<br>days 1, 8, 15<br>+<br>Bevacizumab 10 mg/m^2<br>days 1, 15<br>Q 28 days<br>(B: experimental arm)	Ixabepilone 20 mg/m^2<br>days 1, 8, 15<br>+<br>Bevacizumab 10 mg/m^2<br>days 1, 15<br>Q 28 days<br>(B: experimental arm)	Ixabepilone 20 mg/m^2<br>days 1, 8, 15<br>Q 28 days<br>(A: control arm)

Treatment will continue until progression, death, or prohibitive side effects.

If any patient has a complete response, patient may stop treatment after 2 additional consolidation cycles following documented complete response.

TABLE OF CONTENTS

		PAGE
1.0	OBJECTIVES	5
2.0	BACKGROUND AND RATIONALE	6-10
3.0	PATIENT ELIGIBILITY AND EXCLUSIONS; INCLUSION OF MINORITIES	10-13
4.0	STUDY MODALITIES	14-15
5.0	CORRELATIVE STUDIES	15-16
6.0	TREATMENT PLAN AND ENTRY/REGISTRATION PROCEDURE	16-17
7.0	TREATMENT MODIFICATIONS	17-23
8.0	STUDY PARAMETERS	23-26
9.0	EVALUATION CRITERIA	26-27
10.0	DURATION OF STUDY	27
11.0	STUDY MONITORING AND REPORTING PROCEDURES	28-33
12.0	YALE PRINCIPAL INVESTIGATOR/SPONSOR SAE REPORTING REQUIREMENT	34-37
13.0	MULTI-SITE MANAGEMENT AND COORDINATION	37-39
14.0	STATISTICAL CONSIDERATIONS	40-43
15.0	BIBLIOGRAPHY	44-49
16.0	APPENDICES	
	APPENDIX A- FIGO Staging Criteria	50
	APPENDIX B- Performance Status Criteria	51
	APPENDIX C- Specimen Shipping Instructions	52
17.0	SUGGESTED PATIENT INFORMATION/INFORMED CONSENT	

1.0 OBJECTIVES

1. Primary objective:
   1.1. To assess the activity of ixabepilone with bevacizumab compared to ixabepilone alone in patients with recurrent or persistent platinum-resistant/refractory epithelial (non-mucinous) ovarian, fallopian tube, or primary peritoneal cancer. We will assess this by comparing the ixabepilone +bevacizumab (experimental) arm to the ixabepilone-alone (control) arm for an improvement in median progression free survival (PFS).

2. Secondary objectives:
   2.1. To compare the experimental arm to the control arm for increases in objective response rate (ORR) and durable disease control rate (DDCR).
   2.2. To compare the experimental arm to the control arm for an increase in overall survival (OS).
   2.3. To assess the safety profile of ixabepilone in combination with bevacizumab in ovarian, fallopian tube, or primary peritoneal cancer patients.
   2.4. To assess whether prior treatment with bevacizumab impacts future response to bevacizumab in combination with ixabepilone.

3. Exploratory/correlative objectives:
   3.1. To characterize number, length and composition (e.g., class III β-tubulin expression) of microtentacles (McTNs) isolated from circulating tumor cells isolated from whole blood of patients undergoing treatment with ixabepilone with or without bevacizumab, and correlate with best response, PFS, and OS.
   3.2. To observe McTNs on circulating tumor cells in blood using a novel polyelectrolyte multi-layer (PEM) tethering technology.
   3.3. To correlate *ex vivo* response of McTNs to drug treatment with clinical response in order to develop a real-time assay to predict response to therapy.
   3.4. To explore use of circulating tumor (ct) DNA as a biomarker for disease response and compare its performance to CA-125.
   3.5. To examine whether clinical response to ixabepilone with or without bevacizumab differs between high and low expressors of class III β-tubulin.

2.0 BACKGROUND AND RATIONALE

2.1 Clinical Objectives

In the United States, ovarian, fallopian tube, or primary peritoneal cancer represents the leading cause of death from gynecologic malignancy. In 2016, there will be a projected 22,290 new cases and 14,240 resultant deaths in the United States alone.[1] Due in part to a lack of adequate screening tests and non-specific symptoms, >65% of ovarian, fallopian tube, or primary peritoneal cancers are diagnosed at an advanced stage (III or IV).[2] While the initial response rate following surgical debulking to first-line adjuvant therapy with carboplatin and paclitaxel is as high as 77-90%,[3,4] the majority of patients with a complete pathologic response eventually recur.[5]

Chemosensitivity of recurrent disease is traditionally classified by the interval between the administration of last platinum and diagnosis of recurrence. Tumors are considered *platinum sensitive* if they recur ≥ 6 months after last platinum exposure, *platinum-resistant* if they recur < 6 months after last platinum exposure, and *platinum-refractory* if they progress during platinum therapy.[6] Response rates to re-treatment with platinum after no treatment for two years can be as high as 70%,[7] compared to a rate of 10-40% in platinum-resistant population.[8] Use of non-platinum therapy in the latter produces objective response rates of 10-35% and disease stabilization in an additional 35-50%.[8] Unfortunately, duration of response rarely exceeds 8 months. ***Defining novel chemotherapeutic approaches for the platinum-resistant ovarian cancer population is crucial and therefore a primary goal of this study.***

In 2014, the AURELIA trial[9] demonstrated that addition of bevacizumab, an anti-angiogenic monoclonal antibody against vascular endothelial growth factor (VEGF), to standard cytotoxic chemotherapy statistically improves overall response rate (ORR) and progression-free (PFS) in platinum-resistant disease. In this phase III study, 361 patients were randomized to receive intravenous cytotoxic chemotherapy (weekly paclitaxel 80 mg/m^2 days 1, 8, 15, and 22 of a 28-day cycle, pegylated liposomal doxorubicin 40 mg/m^2 every 4 weeks, topotecan 4 mg/m^2 days 1, 8, 15 of a 21-day cycle or 1.25 mg/m^2 days 1-5 of a 21-day cycle) with or without bevacizumab (10 mg/kg every 2 weeks or 15 mg/kg every 3 weeks). Median PFS was 6.7 versus 3.4 months among patients who did and did not receive bevacizumab, respectively ($p<0.001$, HR 0.42, 95% CI 0.32-0.53). ORR was 27.3% versus 11.8% ($p=0.001$). Median OS did not differ between groups: 16.6 versus 13.3 months ($p=0.174$, HR 0.85, 95% CI 0.66-1.08). Weekly paclitaxel may be synergistic with bevacizumab, as paclitaxel treatment has been shown to induce VEGF expression *in vitro* through the production of reactive oxygen species.[10] Co-administration of bevacizumab may also normalize the tumor vasculature to allow increased delivery of drug.[10] Indeed, early in the AURELIA trial, the weekly paclitaxel cohort appeared to benefit most (PFS 10.4 versus 3.9 months).[11]

At present, bevacizumab is currently approved for use in platinum-resistant recurrent epithelial ovarian, fallopian tube, or primary peritoneal cancer in combination with chemotherapy. In phase II studies by the Gynecologic Oncology Group (GOG 170D), clinical response rate of bevacizumab monotherapy in recurrent epithelial ovarian cancers is approximately 21% (15% complete, 85% partial), with a median PFS and OS of 4.7 and 17 months respectively.[12] Treatment in the primary setting has not gained approval, though bevacizumab has been well-studied in two large phase III

studies. In GOG 218, 1,873 women with stage III or IV previously untreated ovarian cancer were randomized after cytoreduction to adjuvant therapy in one of the three arms: control (carboplatin area under the concentration [AUC] 6 plus paclitaxel 175 mg/m2 plus placebo), control with bevacizumab 15 mg/kg, or control with bevacizumab followed by bevacizumab maintenance.[13] Bevacizumab maintenance therapy resulted in an improved PFS (median PFS 14.1 months vs 10.3 months without bevacizumab, HR 0.717, 95% CI 0.625–0.824, *P*=0.001), but there was no difference in overall survival. In ICON7, 1528 women stage I–IIA high-grade or clear cell ovarian carcinoma or IIB–IV epithelial ovarian cancer, primary peritoneal cancer, or fallopian tube cancer were randomized to first-line adjuvant therapy with carboplatin (AUC 5 or 6) plus paclitaxel 175 mg/m2 every 3 weeks for six cycles or this regimen plus bevacizumab 7.5 mg/kg given concurrently, some of whom received 12 cycles of bevacizumab maintenance.[14] PFS was higher with bevacizumab therapy (median PFS 19.8 months vs 17.4 months, HR 0.87, 95% CI 0.77–0.99, *P*=0.04), without effect on overall survival. It is currently unknown whether prior treatment with bevacizumab alters response to re-treatment. Retrospective studies in recurrent ovarian cancer have suggested improved progression-free survival in bevacizumab-naïve patients who receive bevacizumab with chemotherapy relative to those who previously received bevacizumab.[15] A phase III trial (MITO16MANGO2b) is onging to assess whether administering bevacizumab in combination with chemotherapy in second-line therapy to patients with recurrent ovarian cancer who have received first-line bevacizumab will be more effective than chemotherapy alone **(**NCT01802749),[16] which has been shown for colorectal carcinoma (HR 0.81, p = 0.0062).[17]

Paclitaxel exerts its anti-neoplastic effects through hyperstabilization of microtubules, which are critical for cell division.[19,20] Microtubules consist of eight α- and seven β-tubulin isotypes that form heterodimers and have the ability to rapidly lengthen and contract.[21] The β-tubulin subtypes differ in amino acid composition at the carboxy terminus[22] and possess specific tissue and developmental distributions. Class I represents the constitutively expressed subtype of β-tubulin.[23] Unlike platinum resistance, a prolonged taxane-free interval is <u>not</u> associated with increased response to paclitaxel.[24] Many mechanisms contributing to paclitaxel resistance have been proposed, including altered drug metabolism,[25] overexpression of the drug efflux transporter p-glycoprotein,[26] and changes in activation of apoptosis pathways involving phosphorylation of bcl-2 and bcl-XL.[27] Perhaps among the most important processes contributing to taxane resistance is upregulation of class III β-tubulin, which decreases the binding affinity of paclitaxel.[28,29] We have shown previously that upregulation of class III β-tubulin is linked to paclitaxel resistance and poor survival (**Figure 1**) in a variety of aggressive female cancers, including uterine serous carcinoma[30] ovarian clear cell carcinoma,[31] high-grade serous ovarian carcinoma,[32] and gynecologic carcinosarcoma.[33]

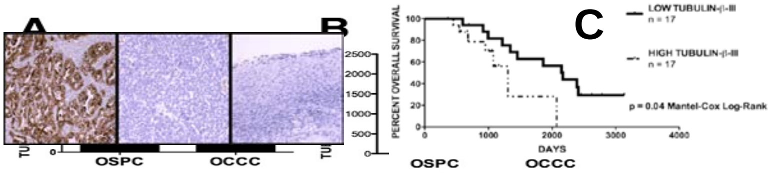

D

Figure 1. Aggressive subtypes of ovarian cancer such as clear cell carcinoma (OCCC) overexpress class III β-tubulin relative to ovarian serous papillary carcinoma (OSPC) in **(A)** fresh frozen tissues (OCCC n= 26; OSPC n=35) and **(B)** cell lines (OCCC n=4; OSPC n=7) as assessed by qRT-PCR. Mean ± SEM are shown. **(C)** qRT-PCR copy number correlates with immunohistochemistry. Representative slides are

shown at original magnification demonstrating OCCC (left, 400x, score 3+) in comparison to OSPC (middle, 400x, score 0+). Corresponding class III β-tubulin expression levels by qRT-PCR in these specimens are 247.83 and 31.55, respectively. Normal ovary (right, 200x, score 0+) does not overexpress class III β-tubulin **(D)** Using qRT-PCR copy number, class III β-tubulin prognosticates poor overall survival in optimally debulked patients with OCCC and OSPC treated with adjuvant carboplatin and paclitaxel chemotherapy.[25]

Epothilones such as ixabepilone (Ixempra,® R-Pharm US, Inc., Princeton, NJ) are microtubule-stabilizing agents isolated from the myxobacterium *Sorangium cellulosum* that are structurally distinct from paclitaxel. Putatively, these agents overcome paclitaxel resistance conferred by the upregulation of class III β-tubulin through equal binding affinity for both class I and class III isoforms.[34] Intracellular concentrations are also better maintained, since epothilones do not serve as a substrate for the p-glycoprotein efflux pump.[35] Ixabepilone has been FDA-approved since 2007 for metastatic or locally advanced breast cancers (1) in combination with capecitabine in anthracycline and taxane-resistant disease and (2) as monotherapy after failure of anthracyclines, taxanes, and capecitabine.[36] ***Ixabepilone has been under-studied for gynecologic indications, but has a number of promising applications.*** In a phase II study of platinum-resistant ovarian cancer by the Gyencologic Oncology Group (GOG 126M),[31] weekly ixabepilone monotherapy at 20 mg/m^2 over 1 hour on days 1, 8, and 15 of a 28-day cycle produced an objective response rate of 14.3% with 3 complete and 4 partial responses and disease stabilization in 40.8%; median progression-free and overall survival was 4.4 and 14.8 months, respecitvely.[38] *There was no assessment of biologic tumor characteristics, and most* ***existing studies of epothilones are flawed in that they lack description of class III β-tubulin status.*** In the current protocol, we will stratify patients by prior receipt of bevacizumab prior to randomization. In order to better understand the association of class III β-tubulin with response to therapy with ixabepilone an exploratory IHC analysis of tumor will be performed. In the chemotherapy-naïve setting, class III β-tubulin expression by IHC occurs in at least 30% of ovarian cancer cells;[39] we expect this number to be higher in the recurrent setting due to development of drug resistance.

In vivo, bevacizumab exhibits greater synergistic antitumor activity in combination with ixabepilone than with paclitaxel,[40] possibly due to greater direct anti-angiogenic activity or alternate pathways to apoptosis.[41] We have previously published our clinical retrospective experience with this combination.[42] Among 60 patients representing 24 uterine and 36 ovarian cancers, patients who received biweekly bevacizumab with weekly ixabepilone exhibited increased progression-free survival (6.5 versus 3.0 months, p=0.01, HR 0.2, 95% CI 0.05-0.77) relative to those who received ixabepilone alone. Patients in this report represented a relatively heavily pre-treated and had received a median of 3.5 (range: 1-10) prior lines of chemotherapy. Toxicities were acceptable, and patients were able to complete a mean of 4.7 ± 2.9 cycles of ixabepilone. ***These findings strongly suggest that this therapeutic combination warrants additional prospective study, and is therefore the subject of this trial.***

2.2 Translational Objectives

2.2.1 Chemosensitivity Assays Using Circulating Tumor Cell Microtentacles

There is a need to develop reliable assays to predict chemosensitivity for recurrent disease. In studies pioneered by the laboratory of Stuart Martin, PhD, at the University of Maryland, microtubule-based extensions of the plasma membrane known as microtentacles (McTNs) have been identified on cells with high metastatic potential in response to various stimuli (**Figure 2**).[43,44,45,46]

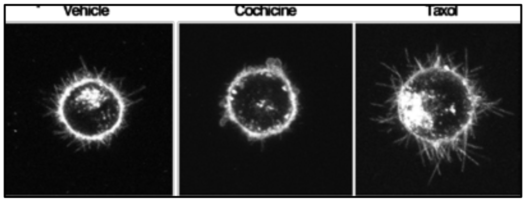

Figure 2. Left: Provoked formation of McTNs (white arrow) in breast cancer cell line SkBr3 by addition of actin in conjunction with paclitaxel. **Middle:** Microtentacle responses to tubulin depolymerization (colchicine) and stabilization (paclitaxel) agents relative to control (vehicle). **Right:** Polyelectrolyte multilayer platform for study of detached cells enhances image quality by eliminating blurring over time to allow for precise measurement of McTN parameters. ***Images courtesy of Stuart Martin, PhD.***

McTNs may be critically linked to metastasis by promoting clustering of free-floating tumor cells as well as adherence to endothelial cells. Very recently, a novel platform consisting of polyelectrolyte multi-layer (PEM) films has been developed through the laboratory of Christopher Jewell in the Department of Bioengineering at the University of Maryland to allow the three-dimensional study of free-floating tumor cells[47] such as those found in ascites or blood, in near-native form. The PEM platform eliminates blurring during confocal microscopy to permit dynamic measurements of McTNs over time (**Figure 3**). This technology would also allow for rapid testing of the effects of drugs on McTNs in as little as a few hours.

One translational component of this study will focus upon the ability to isolate circulating tumor cells from whole blood of patients undergoing treatment with ixabepilone with or without bevacizumab, and explore the possibility to use the patent-pending PEM tethering technology to characterize McTNs. McTN composition (e.g., class III β-tubulin expression), number, and length will be correlated with best response, progression-free survival, and overall survival. We will associate the *ex vivo* responses of McTNs to drug treatment with observed clinical response in order to develop a meaningful real-time assay to predict response to this therapy.

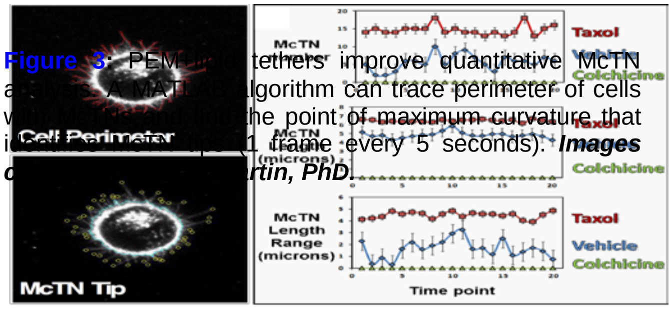

Figure 3: PEM+lipid tethers improve quantitative McTN analysis. A MATLAB algorithm can trace perimeter of cells with McTNs and find the point of maximum curvature that identifies McTN tips (1 frame every 5 seconds). ***Images c[illegible]artin, PhD.***

2.2.2 Circulating Tumor DNA as a Novel Biomarker

Serum CA-125 is the only approved test for monitoring of ovarian cancer response to therapy and disease recurrence.[48] Use of CA-125 for epithelial ovarian cancers was first described in 1983,[49] but exhibits poor performance in pre-menopausal women (sensitivity 50-74%, specificity 69-78%)[50] and

early stage disease, and may not be consistently produced by certain tumors, such as mucinous and clear cell carcinomas as well as carcinosarcomas.[51] Up to 50% of patients with ovarian cancer with normal CA-125 levels may actually have persistent disease.[52]

Circulating tumor (ct) DNA represents a promising method for non-invasive screening and oncologic surveillance.[53] ct-DNA is present in the blood as small DNA fragments released from both primary and metastatic tumors.[54] The measurement of ctDNA has been explored in non-gynecologic malignancy including breast,[55] lung,[56] colon,[57] and gastric carcinoma.[58] In gynecologic cancers, including ovarian, uterine, fallopian tube, and primary peritoneal, preliminary reports[59] have suggested that droplet digital polymerase chain reaction (PCR) for tumor-specific mutations can be used to quantify changes in ct-DNA with good radiologic and biomarker correlation in the primary treatment setting. Relative to traditional PCR platforms, droplet digital PCR offers (1) superior sensitivity, with the ability to detect 1 mutant amongst 250,000 wild-type molecules with a lower limit of detection of 1 in more than 1,000,000 and (2) the capacity for multiplexing by which up to 10 tests or more can be conducted on the same sample using the single molecule multi-color detection technique[60] Additional study of this technology for use in recurrent ovarian cancer is warranted. In the Cancer Genome Atlas Project, 96% of high-grade serous ovarian carcinomas exhibited *TP53* mutations.[61] We will therefore test the performance of *TP53*-mutated ct-DNA as a candidate biomarker for disease status by assessing levels at baseline and throughout treatment, and correlate with CA-125, radiologic response, and PFS/OS.

3.0 PATIENT ELIGIBILITY AND EXCLUSIONS

3.1 Eligible Patients

1. Patients must have platinum-resistant/refractory (i.e., platinum-free interval <6 months) recurrent or persistent histologically confirmed epithelial (non-mucinous) ovarian, fallopian tube, or primary peritoneal cancer.

   Patients may have serous, endometrioid, clear cell, carcinosarcoma, or transitional cell/malignant Brenner, mixed, or undifferentiated histologies.

2. Patients must have specimen available for immunohistochemistry for class III β-tubulin status; recurrent tumor specimen is preferred, though this may be performed on primary tumor if no recurrent tumor is available.

3. All patients must have measurable disease. Measurable disease is defined as lesions that can be measured by physical examination or by means of medical imaging techniques. Measurable disease is defined as at least one lesion that can be accurately measured in at least one dimension (longest dimension to be recorded). Each lesion must be ≥ 20 mm when measured by conventional techniques, including palpation or plain x-ray, or ≥ 10 mm when measured by spiral CT and/or MRI. Ascites and pleural effusions are not to be considered measurable disease.

4. Patients must have at least one "target lesion" to be used to assess response on this protocol as defined by RECIST v1.1.[62] Tumors within a previously irradiated field will be designated as "non-target" lesions unless progression is documented or a biopsy is obtained to confirm persistence at least 90 days following completion of radiation therapy.

5. At the time of initial surgery, patients may have been optimally (<1 cm diameter residual disease) or sub-optimally (≥1 cm diameter of residual disease) debulked.

6. Patients with measurable recurrent disease of any previous stage (I-IV) are eligible to enrollment.

7. The diagnosis must be histologically confirmed by a gynecologic pathologist.

8. Patients must have adequate bone marrow, kidney, and liver function:

   (a) Absolute neutrophil count greater than or equal to 1500 cells/mm^3
   (b) Platelets greater than or equal to 100,000/uL
   (c) Renal function: creatinine less than or equal to 2.0 mg/dL
   (d) Hepatic function: Bilirubin ≤ 1.5 X laboratory normal
   (e) SGOT/SGPT ≤ 3 X laboratory normal.

9. Patients must have an ECOG performance status of 0-2.

10. Patients must have signed an approved informed consent.

11. Patients must have recovered from effects of recent surgery, radiotherapy, or chemotherapy. They should be free of significant infection.

12. Patients must have received prior treatment with paclitaxel (at least 3 cycles). There is no limit on the number of prior lines of therapy.

13. Patients may have received prior bevacizumab therapy alone or in combination with chemotherapy. A 3-week washout period is required.

14. Patients of childbearing potential must have a negative serum pregnancy test within 7 days prior to the study entry and be practicing an effective form of contraception (section 7.5.3).

15. Patients must be at least 18 years of age.

3.2 Ineligible Patients:

1. Patients with a history of other invasive malignancies, with the exception of non-melanoma skin cancers, are excluded if there is any evidence of other malignancy

present within the last five years. Patients are also excluded if their previous cancer treatment contraindicates this protocol therapy.

2. Patients who have a significant history of cardiac disease, i.e., uncontrolled hypertension, unstable angina, uncontrolled congestive heart failure, or uncontrolled arrhythmias within 6 months of registration (NYHA classification III-IV).

3.2.3 Patients with any unstable medical issue (including cardiac issues as above, active treatment for symptomatic pulmonary embolism, CVA, renal or hepatic insufficiency, active infection/sepsis requiring intravenous antibiotics). In patients who have undergone surgery, 28 days should elapse before initiation and the surgical site should be adequately healed.

3.2.4 Known brain/leptomeningeal involvement of the disease, active neurological disease such as uncontrolled seizure disorder or moderate to severe dementia.[57]

3.2.5 Patients who have received prior therapy with any covalent irreversible anti-angiogenic tyrosine kinase inhibitor (e.g., vandetanib).

3.2.6 Patients known to be seropositive for human immunodeficiency virus (HIV) and active hepatitis, even if liver function studies are in the eligible range.

3.2.7 Known hemorrhagic diathesis or active bleeding disorder, including platelet count <100,000/uL, or inadequate granulocytes, including an absolute neutrophil count <1500 cells/mm.[3]

3.2.8 Any hypersensitivity to Cremophor® EL or polyoxyethylated castor oil.

3.2.9 CTCAE grade 2 or higher peripheral neuropathy.

3. Inclusion of Minorities/Rare Tumor Types:

3.1. As men do not have uteri, only women will be enrolled in this trial. Participating institutions will not exclude potential subjects from participating in this or any study solely on the basis of ethnic origin or socioeconomic status. Every attempt will be made to enter all eligible patients into this protocol and therefore address the study objectives in a patient population representative of the entire platinum-resistant/refractory recurrent/persistent ovarian, fallopian tube, or primary peritoneal cancer population treated by participating institutions.

3.3.2 The expected frequency of histologies is serous (80-85%), endometrioid (<10%), clear cell (<5%), carcinosarcoma (<7%), or transitional cell/malignant Brenner (<0.5%), mixed, or undifferentiated histologies. Increasingly, mucinous disease is felt to represent a separate entity, and is therefore not included in this study.[64,65,66]

4.0 STUDY MODALITIES

4.1 Ixabepilone

4.1.1 Preparation

Ixabepilone will be supplied in 15 mg (8 mL) and/or 45 mg (23.5 mL) aliquots with diluent for intravenous administration. The following infusions have been qualified for use in the dilution of ixabepilone:

- Lactated Ringer's
- 0.9% sodium chloride, pH-adjusted with sodium bicarbonate; when using a 250 mL or 500 mL bag of 0.9% sodium chloride injection to prepare the infusion, the pH must be adjusted to 6.0-9.0 by adding 2mEq (i.e., 2 mL of an 8.4% w/v solution or 4 mL of a 4.2% w/v solution) of sodium bicarbonate injection, prior to the addition of the constituted ixabepilone solution
- PLASMA-LYTE pH 7.4

A new bottle of medication will be dispensed on each day of treatment, as once diluted with infusion fluid the solution is stable at room temperature for 6 hours.

To minimize the chance of occurrence of a hypersensitivity reaction, all patients must be premedicated approximately 1 hour before the infusion of ixabepilone with

• An H1 antagonist (eg, diphenhydramine 50 mg orally or equivalent) and

• An H2 antagonist (eg, ranitidine 150 - 300 mg orally or equivalent).

Patients who have experienced a hypersensitivity reaction to ixabepilone require premedication with corticosteroids (e.g., dexamethasone 20 mg intravenously, 30 minutes before infusion or orally, 60 minutes before infusion) in addition to pretreatment with H1 and H2 antagonists.

4.1.2 Storage Conditions

Ixabepilone must be stored at 2-8 ° C protected from light.

4.1.3 Drug Accountability

Drug supplies, which will be provided by R-Pharm US, Inc., will be kept in a secure, limited-access storage area under the above storage conditions. Where necessary, a temperature log must be maintained to make certain that the drug supplies are stored at the correct temperature.

The responsible person must maintain records of the product's delivery to the study site, the inventory at the site, the use by each patient, and the return to the R-Pharm US, Inc. or alternative disposition of unused product(s).

These records will include dates, quantities, batch/serial numbers, expiry ('use by') dates, and the unique code numbers assigned to the investigational product(s) and study patients. The

responsible person will maintain records that document adequately that the patients were provided the doses specified by the study protocol and reconcile all investigational product(s) received from R-Pharm US, Inc. The responsible person must verify that all unused or partially used drug supplies have been returned by the clinical study patient.

4.2 Bevacizumab

4.2.1 Preparation
Bevacizumab will be supplied in 100 mg/4mL or 400 mg/16mL single-use vials (contains no preservative). A new bottle of medication will be dispensed on each day of treatment. Once diluted with infusion fluid the solution is stable at 2-8° C for up to 8 hours.

4.2.2 Storage Conditions
Bevacizumab must be stored at 2-8 °C protected from light. Do not freeze or shake.

4.2.3 Drug Accountability
Drug supplies will be kept in a secure, limited-access storage area under the above storage conditions. Where necessary, a temperature log must be maintained to make certain that the drug supplies are stored at the correct temperature.

The responsible person must maintain records of the product's delivery to the study site, the inventory at the site, the use by each patient, and appropriate disposition of unused product(s) as per institutional guidelines.

These records will include dates, quantities, batch/serial numbers, expiry ('use by') dates, and the unique code numbers assigned to the investigational product(s) and study patients. The responsible person will maintain records that document adequately that the patients were provided the doses specified by the study protocol.

5.0 CORRELATIVE/TRANSLATIONAL STUDIES

5.1 Immunohistochemistry (IHC):

5.1.1 Technical Specifications
Primary or metastatic tumor sites for IHC analysis will have been collected at the time of initial staging surgery, interval debulking, or biopsy-proven recurrence. Archival tumor tissue or tumor cells isolated from ascites can be studied in patients with recurrent disease that do not undergo secondary surgery or biopsy. The tumor will be processed and paraffin-embedded by a research technician in the surgical pathology department. All IHC and H&E will be centrally performed and reviewed in the Pathology Department at Yale University. Patients will be allowed to initiate therapy prior to central pathology confirmation. Sections will be cut at 4 microns and stained with anti-class III β-tubulin monoclonal antibody (TUJ1; Covance, Berkeley CA) at 1:500 dilution with the appropriate positive and negative controls.[30,31,67]

5.1.2 Scoring

Staining of tumor will be according to the following scoring system:[30,31]

0, negative (no staining observed)
1+, focal, weak staining
2+, diffuse weak or focal moderate staining
3+, diffuse moderate or focal strong staining
4+, diffuse, strong immunoreactivity.

5.2 Exploratory Objectives

5.2.1 Changes in Microtentacle Characteristics During Treatment
With the collaborative expertise of Stuart Martin, PhD, circulating tumor cells will be isolated and enumerated from whole blood collected at baseline and every cycle thereafter. If viable cells are isolated, they will be suspended using a polyelectrolyte multi-layer (PEM) tethering technology. Cells will be characterized by class III β-tubulin expression. We will examine whether differences exist over time between patients undergoing treatment with ixabepilone monotherapy versus ixabepilone with bevacizumab. Characteristics will be correlated with best response, PFS, and OS.

5.2.2 Development of Real-Time Chemosensitivity Assay
Floating e*x vivo* viable circulating tumor cells may be exposed to ixabepilone, paclitaxel, and bevacizumab. The observed response of McTNs to treatment will be correlated with clinical response to assess whether this platform can serve as a near real-time assay to predict response to therapy.

5.2.3 Circulating Tumor (ct) DNA as a Biomarker for Disease Response
TP53-mutated ct-DNA isolated from blood will be assessed as a candidate biomarker for disease status as quantified by digital droplet PCR technology. Changes over the course of treatment will be correlated with CA-125, clinical response, and radiologic response, as well as PFS/OS.

5.2.4 Class III β-tubulin Expression and Response to Ixabepilone
Best response, PFS, and OS will be compared between high and low expressors of class III β-tubulin in order to explore its utility as a biomarker for response to ixabepilone

6.0 TREATMENT PLAN AND ENTRY PROCEDURE/REGISTRATION PROCEDURE

6.1 Study Entry

6.1.1 Patients will be screened and consented by the P.I. or a designated member of the study staff at each respective institution.

6.1.2 Treatment Plan
This is a randomized phase II design in patients with platinum-resistant/refractory recurrent/persistent epithelial (non-mucinous) ovarian, fallopian tube, or primary peritoneal cancer. A treatment cycle is defined as 28 days. Study drug will be prescribed by the Principle

Investigator/Sub-Site Principal Investigator and may be dispensed either by the investigator, site staff or affiliated pharmacy. Treatment may continue indefinitely until progression, death, or intolerable side effects. In the event of a complete response, patients may discontinue therapy after 2 consolidation cycles following documented complete response. Such patients may undergo a second course of treatment/re-treatment within the study design.

6.1.3 Methods of Chemotherapy Administration
Maximum body surface area used for dose calculations will be 2.0 m^2 as per GOG Chemotherapy Procedures Manual.

6.1.4 Sequence and Timing of Drug Administration
Ixabepilone will be administered at 20 mg/m^2 intravenously days 1, 8, 22 of a 28-day cycle in Lactated Ringers' solution (concentration 0.2 to 0.6 mg/mL) over one hour.[68] If assigned to receive bevacizumab at 10 mg/kg days 1 and 15 of a 28-day cycle, bevacizumab will be infused after ixabepilone. The first dose of bevacizumab will be administered intravenously in 0.9% NaCl 100 mL over 90 minutes; the second dose may be administered over 60 minutes if no prior reaction to previous infusion; subsequent doses may be administered over 30 minutes if no prior reaction to previous infusion. Bevacizumab should NOT be administered or mixed with dextrose or glucose solutions. A concentration-dependent degradation profile of bevacizumab was observed when diluted with dextrose solutions (5%).

7.0 TREATMENT MODIFICATIONS

7.1 Dose Reduction for Ixabepilone

7.1.1 To prevent the development of more severe adverse events (AE), treatment-related side effects should be managed early and proactive as described in the Table below.

Table 1: Dose reduction scheme for ixabepilone

**AE type and CTCAE Grade**[1]	**Dose reduction scheme**
NEUROLOGIC	
Grade 2 neuropathy lasting ≥ 7 days	Decrease dose by 20%
Grade 3 neuropathy lasting < 7 days	Decrease dose by 20%
Grade 3 neuropathy lasting ≥ 7 days	Discontinue treatment[2]
Any grade 3 toxicity other than neuropathy	Decrease dose by 20%
Transient grade 3 arthralgia/myalgia or fatigue	No change in dose
Grade 3 hand-foot syndrome (palmar plantar erythrodysethsia)	No change in dose
Any grade 4 toxicity	Discontinue treatment

HEMATOLOGIC<br><br>Neutrophil < 500 cell/mm^3 for ≥ 7 days<br>Febrile neutropenia<br>Platelets < 25,000/mm or platelets <50,000/mm with bleeding	 <br>Decrease dose by 20%<br>Decrease dose by 20%<br>Decrease dose by 20%
HEPATIC: avoid concomitant CYP3A4 inhibitors/inducers<br><br>AST and ALT ≤2.5 times ULN and bilirubin ≤1 times ULN<br><br>AST and ALT >2.5 to ≤10 times ULN and bilirubin >1 to ≤1.5 times ULN<br><br>AST and ALT ≤10 times ULN and bilirubin >1.5 to ≤3 times ULN<br><br>AST or ALT >10 times ULN or bilirubin >3 times ULN	 <br>No dosage adjustment necessary<br><br>Decrease dose by 20%<br><br>Decrease dose by 50%; may escalate up to a dose reduction of 25%<br><br>Discontinue treatment
RENAL: there are no dose adjustments for renal function	

[1] Baseline is defined as the CTCAE (version 4.0) Grade at the start of treatment.
[2] In the event that the patient is deriving obvious clinical benefit according to the investigator's judgement, further treatment with will be decided in agreement between the sponsor and the investigator.
ULN = upper limit of normal

In the event of any unrelated adverse events, the Principal Investigator/Sub-Site Principal Investigator may choose to interrupt the medication for up to 14 days, but no dose reduction should occur. If the medication is interrupted for more than 14 days, the decision to continue with treatment will be made by the treating investigator.

7.1.2 See package insert for further details.

7.2. Dose Reduction for Bevacizumab

7.2.1 There are no recommended dose reductions for bevacizumab, however, drug should be suspended in the following situations:

- At least 4 weeks prior to elective surgery
- Severe hypertension not controlled with medical management
- Moderate to severe proteinuria
- Severe infusion reactions

discontinued in the following situations:

- Gastrointestinal perforation, internal organ/trachea-esophageal fistula, or other grade 4 fistulae
- Wound dehiscence or healing complications
- Hemorrhage requiring medical interventions
- Severe arterial thrombosis or grade 4 venous thromboembolism or pulmonary embolus
- Posterior Reversible Encephalopathy Syndrome [PRES]
- Nephrotic syndrome: monitor with dipstick; patients with 2+ or greater urine dipstick reading should undergo a 24-hour urine collection (NOT urine protein:creatinine ratio); treatment should only be given when proteinuria is <2g/24 hours.

7.2.2 See package insert for further details.

7.3 Rescue Medication, Emergency Procedures, and Concomitant Treatments

7.3.1 Rescue Medication

7.3.1.1 Ixabepilone

Rescue medications to reverse the actions of ixabepilone are not currently available. There is no specific antidote for over dosage with ixabepilone. The adverse reactions reported in these cases included peripheral neuropathy, fatigue, musculoskeletal pain/myalgia, and gastrointestinal symptoms (nausea, anorexia, diarrhea, abdominal pain, stomatitis). The highest dose mistakenly received was 100 mg/m^2 (total dose 185 mg). Potential adverse events should be treated symptomatically.

7.3.1.2 Bevacizumab

Rescue medications to reverse the actions of bevacizumab are not currently available. The highest dose of bevacizumab tested in humans (20 mg/kg of body weight, intravenous, multiple doses) was associated with severe migraine in several patients.

7.3.2 Emergency Procedures

Ixabepilone and bevacizumab will usually be administered in an outpatient setting. When study medication is administered to a patient, emergency resuscitation equipment must be available in the clinic. Patients must remain under medical supervision for 1 hour following completion of therapy on day 1 of the first cycle.

7.3.3 Concomitant Treatment(s)

Concomitant medications or therapy to provide adequate supportive care may be given as clinically necessary.

After study enrollment, palliative radiotherapy may be given for bone pain or for other reasons (e.g. bronchial obstruction, skin lesions), provided that the total dose delivered is in a palliative

range according to institutional standards. The irradiated area cannot be used for tumor response assessment. During palliative radiotherapy, study treatment should be delayed and may be resumed once the patient has recovered from any radiation associated toxicity. If medication is interrupted for more than 14 days, the decision to continue will be made by the Principal Investigator/Sponsor in agreement with the Sub-Site Principal Investigator. Continuous interruption of >28 days due to palliative radiotherapy will not be allowed.

All concomitant therapy, including anaesthetic agents, vitamins, homeopathic/herbal remedies, nutritional supplements, must be recorded in the case report form (CRF) during the screening and treatment period, starting from the date of signature of informed consent, and ending at the end of treatment (EOT) visit. After the EOT visit, only concomitant therapy indicated for treatment of an adverse event has to be reported.

In case of emergent surgery, it is recommended to restart treatment after complete wound healing. If therapy is interrupted for more than 14 days, the decision to continue will be made by the Principal Investigator/Sponsor in agreement with the Sub-Site Principal Investigator.

7.4 Management of Expected Adverse Events

To improve tolerability and the probability of clinical benefit, patients should receive prompt and appropriate supportive care at the first signs of symptoms. Suggested treatments for adverse events are described below.

7.4.1 Gastrointestinal

7.4.1.1 It is anticipated that nausea and vomiting may be a side effect of chemotherapy. The following representative antiemetic regimens are suggested: ondansetron 8-32 mg IV 30 minutes prior to administration of chemotherapy and dexamethasone 10-20 mg IV 30 minutes prior to drug administration, or granisetron 10 mcg/kg IV (or 2 mg PO) 30 minutes prior to chemotherapy, with or without lorazepam 0.5-2.0 mg IV 30 minutes prior to chemotherapy or palonosetron 0.25 mg IV 30 minutes prior to chemotherapy.

7.4.1.2 Patients may receive the treating investigator’s choice of drugs for the outpatient control of nausea/ vomiting. Grade 4 gastrointestinal toxicity will require hospital admission for IV hydration.

7.4.1.3 If any mucositis is present on day 28 of a cycle, treatment should be withheld until the mucositis has returned to Grade 1. Grade 3 or 4 mucositis or diarrhea requires a dose reduction of ixabepilone.

7.4.1.4 Bilirubin must be within normal limits prior therapy.

7.4.2 Hematologic

7.4.2.1 Initial treatment modifications will consist of cycle delay and/or dose reduction as indicated in Section 7.1. The use of hematopoietic cytokines and protective reagents are restricted as follows

- patients will NOT receive prophylactic growth factors [filgrastim (GCSF), sargramostim (GM-CSF), pegfilgrastim (Neulasta)] unless they experience recurrent neutropenic complications after treatment modifications specified above

- patients will NOT receive prophylactic thrombopoietic agents

- patients may receive erythropoietin for management of anemia AFTER documentation of hemoglobin less than 10 g/dl (CTCAE v4.0 grade 2)

7.4.2.2 Treatment decisions will be based on the absolute neutrophil count (ANC) rather than the total white cell count (WBC).

7.4.2.3 Subsequent cycles of therapy will not begin until the ANC is ≥1500 cells/microliter (CTCAE v4.0 grade 1) and the platelet count is ≥ 100,000/microliter. Therapy will be delayed for a maximum of two weeks until these values are achieved. Patients who fail to recover adequate counts within a two week delay will be removed from study therapy.

7.4.2.4 For first occurrence of febrile neutropenia, and/or documented grade 4 neutropenia persisting ≥7 days, reduce ixabepilone chemotherapy by one dose level on subsequent cycles.

7.4.2.5 For recurrent febrile neutropenia, and/or recurrent documented grade 4 neutropenia persisting ≥7 days (after initial dose reduction), add prophylactic growth factors. In this circumstance, it is recommended that G-CSF at a dose of 5 microgram/kg/day* (or equivalent dosing of pegfilgrastim or sargramostim) will be administered subcutaneously starting the day after the last dose of chemotherapy (normally day 2-4) and continuing through hematopoietic recovery. Growth factors should not be used within 72 hours of a subsequent dose of chemotherapy.

7.4.3 Neurotoxicity

Neurologic Toxicity Grade 2 (at the discretion of the treating investigator) or Grade 3 or greater peripheral neuropathy requires discontinuation of protocol therapy until symptoms resolve to Grade 1. The protocol regimen will be discontinued in patients with recurrent Grade 3 or any Grade 4 neurologic toxicity.

7.4.4. Genitourinary

Increased frequency with dysuria may occur in some patients. This may be treated symptomatically with antispasmodics and increased fluid intake. Urinary tract infection should be ruled out if the symptoms persist.

7.4.5 Cardiovascular

7.4.5.1 Asymptomatic bradycardia is not an indication for discontinuation of therapy, however, infusion should be discontinued for chest pain, clinically significant arrhythmia, hypotension,

or serious allergic reaction. Any arrhythmia that is felt to necessitate discontinuation of therapy should be discussed with the Study Chair as soon as possible.

7.4.5.2 Assessment, surveillance, and management of blood pressure is required in patients receiving vascular endothelial growth factor signaling pathway inhibitors. For patients who develop hypertension while receiving treatment with an antiangiogenic agent, treatment with angiotensin system inhibitors (e.g., angiotensin converting enzyme inhibitors [ACEIs], angiotensin receptor blockers [ARBs]) may be preferred over other agents.

Treating investigators should refer to guidelines established for use of antiangiogenic agents:[69]

- identify and treat preexisting hypertension before using these agents
- actively monitor blood pressure during treatment, with more frequent measurement in the first several weeks of therapy
- manage blood pressure during therapy with an ideal goal of <130/80 mmHg for most patients, lower in those with specific preexisting cardiovascular risk factors, such as diabetes or chronic kidney disease
- patients who develop hypertension during treatment (defined as blood pressure ≥140 mm Hg/90 mmHg or a 20 mm increase in diastolic blood pressure over baseline) should be treated with antihypertensives

7.4.6 Hypersensitivity Reaction

7.4.6.1 If hypersensitivity reactions to ixabepilone or its vehicle (Cremaphor) occur, it will usually be during the first few minutes of infusion. Appropriate symptomatic therapy should be given. Continued treatment may be considered if the reaction is not life-threatening; however, patients must be cautioned about potential recurrences of the reaction. Should the patient decide to continue with treatment it is preferable that this be done on the same day of the occurrence. Patients will follow the institutional protocol for desensitization. A suggested procedure would be to administer the drug first with 1 ml of the original IV solution diluted in 100 ml over one hour, then 5 ml in 100 ml over one hour, then 10 ml in 100 ml over one hour, and finally, the original solution at the original speed.

7.4.6.2 There are no data to address routine premedication use or reinstitution of therapy in patients who experience severe infusion reactions to bevacizumab.

7.4.7 Musculoskeletal

In the several days following ixabepilone treatment may be severe, and should receive aggressive symptomatic treatment, including narcotics or steroids as required. They are not, however, an indication for dose reduction.

7.5 Restrictions

7.5.1 Concomitant Treatment

Concomitant medications, or therapy to provide adequate supportive care, may be given as clinically necessary. Additional experimental anti-cancer treatment and/or standard chemo-,

immunotherapy, hormone treatment (with the exception of megestrol acetate)), or radiotherapy (other than palliative radiotherapy for symptom control) is not allowed concomitantly with the administration of study treatment.

7.5.2 Diet and Lifestyle

Patients should be advised to avoid any foods known to aggravate diarrhea/constipation. To prevent skin-related adverse events it is recommended to avoid intense irradiation with UV light and harsh detergents.

7.5.3 Women of Child-Bearing Potential and Pregnancy Prevention

Patients who are not of childbearing potential due to being postmenopausal (1 year without menstruations and at least 2 years without menstruation following chemotherapy) or surgical sterilisation (oophorectomy, hysterectomy and/or tubal ligation) do not need to use contraception to be eligible for the trial. All other patients are considered to have childbearing potential and must use adequate contraception throughout the trial (from screening until end of trial participation).

Acceptable methods of contraception include surgical sterilization and double barrier method, and must be in accordance with local regulations where applicable. Double barrier method of contraception is defined as two barrier methods used simultaneously each time the patient has intercourse. Accepted barrier methods include diaphragm, female condom, cervical cap, male condom and intrauterine device (UID) (the diaphragm and cervical cap must be used in conjunction with spermicidal jelly/cream). Those using hormonal contraceptives, or with partners using hormonal contraceptives, must also be using an additional approved method of contraception (as described above). Partner vasectomy, natural "rhythm," and spermicidal jelly/cream are not acceptable methods of contraception.

Women who become pregnant while participating in the study must discontinue study medication immediately. The pregnancy must be reported following procedures detailed in section 11.10.2.

7.6 Treatment Compliance

The study medication will be given in accordance with the protocol and the instructions of a Sub-Site Principal Investigator.

The Sub-Site Principal Investigator and/or the Principal Investigator/Sponsor can withdraw a patient from the study in the event of serious and persistent non-compliance which jeopardizes the patient’s safety or render study results for this patient unacceptable. Patients who do not attend a minimum of *75%* of scheduled study visits, unless due to exceptional circumstances, should be discussed with the sponsor and be evaluated for compliance.

8.0 <u>STUDY PARAMETERS</u>

8.1 Observations and Tests

The following observations and tests are to be performed and recorded before, during, and after treatment:

Study week	**Pre-Treatment** (-28 to 1 Days)	**Treatment Period** Cycle 1	**Treatment Period** Cycle 2 and higher	**End of treatment visit**
**Cycle Day**		1 8 15	1***	
**Study Procedures Window (days)**		0 ± **3** ± **3**	± **3**	
History, physical exam, performance status, toxicity evaluation,	X	X X X	X	X
CBC with differential including platelets	X	X X X	X	X
Chemistries: serum Cr, BUN, K, Mg, bilirubin	X	X X X	X	X
Urinalysis	X	X --- X	continue urinalysis days 1 and 15	X
SGOT,SGPT,Alk Phos	X	X X X	X	X
Vital signs, weight	X	X X X	X	X
EKG	X	only as clinically indicated****	only as clinically indicated****	X
Toxicity	X	X X X	X	X
Radiographic tumor evaluation and measurement (Chest/Abd/Pelvis)*	X	N/A	X*	X
CA 125	X	N/A	X	X
Collection of blood for translational studies**	X	N/A	X	X

* Repeat CT scan or MRI should use similar equipment and techniques to ensure consistent measurements. Imaging should be performed at baseline and q 2 cycles.

** See Appendix C for collection and shipping information. If progression after 2 cycles (8 weeks) collect at End of Treatment.

*** All activities performed every 4 weeks

.

**** During the treatment period, EKG will be performed on screening and as clinically indicated thereafter.

Screening procedures include:

- A review of the patient's medical history including a list of current medications and dosing schedules, previous therapies for disease, and residual treatment toxicities
- Physical examination, height, weight and vital signs (pulse, temperature, respiration, and blood pressure); a pelvic examination may be required if this method is used to measure patients disease
- An electrocardiogram (ECG) will be performed for baseline assessment
- A CT scan of chest, abdomen and pelvis will be performed or an MRI if necessary; the scans may not have to be repeated if performed within 28 days
- Serum CA 125
- Lab work: CBC, Chemistry (electrolytes, magnesium, SGOT, SGPT, alkaline phosphatase)
- In women of child-bearing potential, a pregnancy test will be performed using either urine or blood

Treatment Procedures include
The following will take place at different times during the treatment period:

- At each study visit (days 1, 8, and 15 of the first cycle, then every 4 weeks) a physical examination including weight and vital signs (pulse, temperature, respirations, blood pressure, weight); pelvic examination may be required if this method is used to measure disease
- Toxicity assessment (weekly during first cycle of treatment)
- Routine lab work such as complete blood count, serum chemistries, and electrolytes will be obtained
- Distribution of study medication, instructions for taking medication, study diary review
- A CT or MRI of the chest abdomen and pelvis will be performed after every 2 cycles of therapy (i.e., after 8 weeks, 16 weeks, etc). CA 125 Protein levels will be drawn at each cycle.
- Approximately 4 teaspoons (30 mL) of blood will be collected for translational studies to assess circulating tumor cells at baseline then every cycle

End-of- Treatment Visit:

- Physical examination will be performed; a pelvic examination is required if this method is used to measure your disease.
- A CT scan of the chest, abdomen, and pelvis will be performed, if not done within prior 3 weeks.
- Serum CA 125 Protein

Follow-up:
Subjects will need to see their doctor every 3 months to check the status of their disease for 2 years following study treatment. Afterwards, they will see the doctor every 6 months for 3 years and then yearly. After disease progression, patients will be followed for survival only every 3 months; this may be performed by telephone follow up.

Note: In order to more precisely determine time of progression, the treating investigator is encouraged to obtain radiologic assessments earlier than 8 weeks if there is a strong clinical suspicion of progression of disease to either confirm or refute the clinical impression.

9.0 EVALUATION CRITERIA

9.1 Objective Response
The major parameters of response to be assessed include PFS, OS, documentation of sites of recurrence, and treatment-related toxicity. Treatment response will be based on RECIST v1.1 Guidelines for measurable lesions.[62] Only recurrent patients with measurable lesions will be enrolled in the study.

9.1.1 Target lesions must be assessed using the same technique as baseline. Included in the evaluations are the following standard response criteria for target lesions:

- Complete Response (CR): Disappearance of all target lesions. No new lesions. Lymph nodes must be <10mm short axis.
- Partial Response (PR): At least a 30% decrease in the sum of the longest diameter of target lesions, taking as reference the baseline sum of the longest diameters. No new lesions.
- Progressive Disease (PD): The appearance of a new lesion, or at least a 20% increase in the sum of the longest diameters of the target lesions, taking as the reference the smallest sum of the longest diameters recorded since treatment started, and at least 5mm increase.
- Stable Disease (SD): Target lesions do not qualify for CR, PR, or progression. No new lesions.

Non-target lesions must be assessed using the same technique as baseline. Included in the evaluations are the following standard response criteria for non-target lesions:

- Complete Response: The disappearance of all non-target lesions.
- Partial Response/Stable Disease: The persistence of one or more non-target lesion(s).
- Progressive Disease: The appearance of one or more new lesions and/or unequivocal progression of existing non-target lesions.

9.2 Progression-Free Survival will be defined as date from entry to a particular protocol to date of reappearance or increasing parameters of disease on imaging (CT scan or MRI) or by clinical exam, or death from any cause, or is censored at date of last disease assessment.

9.3 Overall Survival will be defined as observed length of life from entry to a particular protocol to death OR for living patients to date of last contact.

9.4 Sites of Recurrence will be assessed. Any clinical or radiological evidence for new tumor, preferably confirmed by pathology, will be considered as a recurrence. Pattern of relapse to be noted:

Pelvic (peritoneal, nodal)
Abdominal (peritoneal, nodal, visceral, ascites)
Diaphragmatic
Distant (nodal, parenchymal, liver, lungs, others)
Omental
Effusions (pleural, ascites)

9.5 The grade level of the various toxicities will be classified using the Common Terminology Criteria for Toxicity version 4 (CTCAE v.4) guidelines.[70] *Acute* toxicities will be scored if occurring ≤ 30 days from treatment completion, and *chronic* if > 30 days. Frequency and duration of treatment interruptions due to the treatment toxicity will be assessed.

10.0 <u>DURATION OF STUDY</u>

1. This study will continue as long as treatment protocols remain activated.

10.2 The patient must be followed quarterly for two years and every six months for three additional years, and thereafter annually or at time of recurrence until death.

11.0 STUDY MONITORING AND REPORTING PROCEDURES

11.1 Personnel Responsible for the Safety Review and Its Frequency

The Principal Investigator/Sponsor will be responsible for monitoring the data, assuring protocol compliance, and conducting the safety reviews at the specified frequency, which must be conducted at a minimum of once every 6 months (including when re-approval of the protocol is sought). During the review process, the Principal Investigator/Sponsor with the help of the Remote Monitoring Coordinator will evaluate whether the study should continue unchanged, require modification/amendment, or close to enrollment. The Principal Investigator/Sponsor and the IRB or the Yale Cancer Center Data and Safety Monitoring Committee (DSMC) have the authority to stop or suspend the study or require modifications.

11.2 Risk Assessment

The risks associated with the current study are deemed *moderate* for the following reasons:

1. We do not view the risks associated with the combination of ixabepilone with bevacizumab as *minimal*.
2. Ixabepilone is FDA-approved as monotherapy for metastatic or locally advanced breast carcinoma after failure of an anthracycline, taxane, and capecitabine. Bevacizumab is FDA-approved in metastatic HER2-negative breast cancer and platinum-resistant recurrent ovarian/fallopian tube/primary peritoneal cancer in combination with chemotherapy, therefore we do not view the proposed study as *high risk*.

Although we have assessed the proposed study as one of moderate risk, the potential exists for anticipated and/or unanticipated adverse events, serious or otherwise, to occur since it is not possible to predict with certainty the absolute risk in any given individual or in advance of first-hand experience with the proposed study methods. Therefore, we provide a plan for monitoring the data and safety of the proposed study as outlined below.

11.3 Attribution of Adverse Events

Adverse events will be monitored for each subject participating in the study and attributed to the study procedures / design by the Principal Investigator/Sponsor, Dr. Alessandro Santin, according to the following categories:

a.) Definite: Adverse event is clearly related to investigational procedures(s)/agent(s).
b.) Probable: Adverse event is likely related to investigational procedures(s)/agent(s).
c.) Possible: Adverse event may be related to investigational procedures(s)/agent(s).
d.) Unlikely: Adverse event is likely not to be related to the investigational procedures(s)/agent(s).
e.) Unrelated: Adverse event is clearly not related to investigational procedures(s)/agent(s).

11.4 Plan for Grading Adverse Events:

Grade refers to the severity of the adverse event. The Common Terminology Criteria for Adverse Events (CTCAE) scale will be used to grade the severity of adverse events noted during the study. The CTCAE displays grades 1 through 5 with unique clinical descriptions of severity for each AE based on this general guideline:

1. Grade 1: mild; asymptomatic or mild symptoms; clinical or diagnostic observations only; interventions not indicated.
2. Grade 2: moderate; minimal, local, or non-invasive intervention indicated; limiting age-appropriate instrumental activities of daily living (ADL) (e.g., preparing meals, shopping for groceries or clothes, using the telephone, managing money, etc.)
3. Grade 3: severe or medically significant but not immediately life threatening; hospitalization or prolongation of hospitalization indicated; disabling; limiting self-care ADL (e.g., bathing, dressing and undressing, feeding self, using the toilet, taking medications, and not bedridden).
4. Grade 4: life-threatening consequences; urgent intervention indicated.
5. Grade 5: death related to adverse event (AE).

11.5 Plan for Determining Seriousness of Adverse Events

An adverse event may be graded as severe but still not meet the criteria for a serious adverse event. Similarly, an adverse event may be graded as moderate but still meet the criteria for an SAE. Severity refers to the intensity of the event without regard for whether or not it meets federal criteria for 'serious.' All AEs and SAEs whether volunteered by the subject, discovered by study personnel during questioning or detected through physical examination, laboratory test, or other means will be recorded appropriately.

11.6 Plan for Reporting Adverse Events and Other Unanticipated Problems Involving Risks to Participants or Others to the IRB

The Principal Investigator/Sponsor will report the following types of events to the IRB: a) adverse events that are serious or life-threatening AND unanticipated (or anticipated but occurring with a greater frequency than expected) AND possibly, probably or definitely related to the drug/device/intervention; and b) other unanticipated problems involving risks to subjects or others.

**Toxicity Grade**	**Type[a]**	**Local IRB**	**Study Coordinators Via email/fax/phone**
4,5	Unknown	Yes	Yes
5	Known	No	Yes[*]

2,3	Unknown	No	No
4 (non-myelo)	Known	No	No
4 (myelo[b])	Known	No	No

[*] If clearly related to the commercial agent(s)
[a] Type (known or unknown) is based on toxicities included in the package insert or literature of known toxicities associated with the study drug(s).
[b] Myelosuppression, which includes neutropenia, anemia, thrombocytopenia

These adverse events or unanticipated problems involving risks to subjects or others will be reported to the IRB in accordance with IRB Policy 710, using the appropriate forms found on the website. This information is discussed in Section 12.0 of the protocol.

11.7 Plan for Reporting Adverse Events to Co-Investigators, the Protocol's Research Monitor(s), the Data and Safety Monitoring Committee (DSMC), Protocol Review Committee (PRC), DSMBs, Study Sponsors, Funding/Regulatory Agencies, and Regulatory/Decision-making bodies

For the current study, all Co-Investigators listed on the protocol, the Yale Cancer Center Data and Safety Monitoring Committee (DSMC) and appropriate IRB(s), and R-Pharm US, Inc. will be notified of Adverse Events. As this study incorporates use of approved products for a new indication/new patient population, the investigator is responsible for reporting in accordance with FDA regulations 21CFR 312.32 (a)(IND Safety Reports). Refer further to http://www.fda.gov/downloads/Drugs/GuidanceComplianceRegulatoryInformation/Guidances/UCM227351.pdf.

The Principal Investigator/Sponsor, Alessandro Santin, will conduct a review of all adverse events upon completion of every study subject. The Principal Investigator/Sponsor will evaluate the frequency and severity of the adverse events and determine if modifications to the protocol or consent form are required.

11.8 Adverse Event Reporting
This study will utilize the Common Terminology Criteria for Adverse Event (CTCAE) version 4.0. A copy can be downloaded from the CTEP home page, using the following link: https://evs.nci.nih.gov/ftp1/CTCAE/About.html.

11.9 Assessment of Safety

11.9.1 Specification of Safety Variables
Safety assessments will consist of monitoring all adverse events and reporting adverse events (AEs) and serious adverse events (SAEs) that are considered related to ixabepilone,

bevacizumab, or the combination, all events of death, and any study specific issue of concern.

11.9.2 Adverse Events (AE)

An AE is any unfavorable and unintended sign, symptom, or disease temporally associated with the use of an investigational (medicinal) product or other protocol-imposed intervention, regardless of attribution.

This includes the following:

- AEs not previously observed in the subject that emerge during the protocol-specified AE reporting period, including signs or symptoms associated with advanced recurrent uterine serous papillary carcinoma that were not present prior to the AE reporting period. See Section 11.10.
- Complications that occur as a result of protocol-mandated interventions (e.g., invasive procedures such as cardiac catheterizations).
- If applicable, AEs that occur prior to assignment of study treatment associated with medication washout, no treatment run-in, or other protocol-mandated intervention.
- Preexisting medical conditions (other than the condition being studied) judged by the treating investigator to have worsened in severity or frequency or changed in character during the protocol-specified AE reporting period.

11.9.2 Serious Adverse Events

Refer to Section 12.0 of the study protocol.

11.9.3 Methods and Timing for Assessing and Recording Safety Variables

The Principal Investigator/Sponsor is responsible for ensuring that all AEs and SAEs that are observed or reported during the study, as outlined in Section 11.10 and 12.0 respectively, are collected and reported to the appropriate IRB(s), and R-Pharm US, Inc. in accordance with FDA regulations at 21CFR 312.32 (a)(IND Safety Reports).

11.9.4 Adverse Event Reporting Period

The study period during which all AEs and SAEs must be reported begins after informed consent is obtained and initiation of study treatment and ends 30 days following the last administration of study treatment or study discontinuation/termination, whichever is earlier. After this period, Co-Investigators should only report SAEs that are attributed to prior study treatment.

11.9.5 Assessment of Adverse Events

All AEs and SAEs whether volunteered by the subject, discovered by study personnel during questioning, or detected through physical examination, laboratory test, or other means will be reported as outlined in section 11.6. Any toxicity which meets criteria to be reported to Study Coordinators via email/fax/phone (above, section 11.6) should also be reported to RPHarm-

US.

Each reported AE or SAE will be described by its duration (i.e., start and end dates), regulatory seriousness criteria if applicable, suspected relationship to ixabepilone, bevacizumab, or the combination (see following guidance), and actions taken.

To ensure consistency of AE and SAE causality assessments, the Principal Investigator/Sponsor should apply the following general guidelines:

a.) Definite: Adverse event is clearly related to investigational procedures(s)/agent(s). There is a plausible temporal relationship between the onset of the AE and administration of ixabepilone, bevacizumab, or the combination, and the AE cannot be readily explained by the subject's clinical state, intercurrent illness, or concomitant therapies; AND/OR the AE follows a known pattern of response to ixabepilone, bevacizumab, or the combination and/or the AE abates OR resolves upon discontinuation of ixabepilone, bevacizumab, or the combination OR dose reduction and, if applicable, reappears upon re-challenge.

b.) Probable: Adverse event is likely related to investigational procedures(s)/agent(s).

c.) Possible: Adverse event may be related to investigational procedures(s)/agent(s).

d.) Unlikely: Adverse event is likely not to be related to the investigational procedures(s)/agent(s).

e.) Unrelated: Adverse event is clearly not related to investigational procedures(s)/agent(s). Evidence exists that the AE has an etiology other than ixabepilone, bevacizumab, or the combination (e.g., pre-existing medical condition, underlying disease, intercurrent illness, or concomitant medication); and/or the AE has no plausible temporal relationship to administration of ixabepilone, bevacizumab, or the combination (e.g., cancer diagnosed 2 days after first dose of study drug).

Expected adverse events are those adverse events that are **listed** or characterized in the Package Insert or current Investigator Brochure.

Unexpected adverse events are those **not listed** in the Package Insert (P.I.) or current Investigator Brochure (I.B.) or not identified. This includes adverse events for which the specificity or severity is not consistent with the description in the P.I. or I.B. For example, under this definition, hepatic necrosis would be unexpected if the P.I. or I.B. only referred to elevated hepatic enzymes or hepatitis. **Refer to Section 12.0 for further guidelines**.

The treating investigator will be responsible for performing this assessment, with oversight of the Principal Investigator/Sponsor.

11.10 Eliciting Adverse Events

A consistent methodology for eliciting AEs at all subject evaluation time points should be adopted. Examples of non-directive questions include:

"How have you felt since your last clinical visit?

"Have you had any new or changed health problems since you were last here?"

11.11 Specific Instructions for Recording Adverse Events

Co-Investigators should use correct medical terminology/concepts when reporting AEs or SAEs. Avoid colloquialisms and abbreviations. All information will be recording using Oncore Clinical Trials Management System.

a. Diagnosis vs. Signs and Symptoms

If known at the time of reporting, a diagnosis should be reported rather than individual signs and symptoms (e.g., record only liver failure or hepatitis rather than jaundice, asterixis, and elevated transaminases). However, if a constellation of signs and/or symptoms cannot be medically characterized as a single diagnosis or syndrome at the time of reporting, it is ok to report the information that is currently available. If a diagnosis is subsequently established, it should be reported as follow-up information.

b. Deaths

All deaths that occur during the conduct of the research must be reported to the Principal Investigator/Sponsor and the Remote Monitoring Coordinator immediately (if possible) to ensure that all over site committees are properly informed. All information will be entered into Oncore Clinical Trials Management System and updated appropriately. Further determination regarding the need to submit to the subjects local IRB, Yale University HHRP following IRB Policy 710 for reporting Unanticipated Problems Involving Risk to Subjects and RPharm US Drug Safety Committee will be reviewed. Specific information is outlined in section 12.0 of the protocol.

When recording a death, the event or condition that caused or contributed to the fatal outcome should be reported as the single medical concept. If the cause of death is unknown and cannot be ascertained at the time of reporting, report "Unexplained Death".

c. Preexisting Medical Conditions

A preexisting medical condition is one that is present at the start of the study. Such conditions should be reported as medical and surgical history.

A preexisting medical condition should be re-assessed throughout the trial and reported as an AE or SAE only if the frequency, severity, or character of the condition worsens during the study. When reporting such events, it is important to convey the concept that the preexisting condition has changed by including applicable descriptors (e.g., "more frequent headaches").

d. Hospitalizations for Medical or Surgical Procedures

Any AE that results in hospitalization or prolonged hospitalization should be documented and reported as an SAE.

If a subject is hospitalized to undergo a medical or surgical procedure as a result of an AE, the event responsible for the procedure, not the procedure itself, should be reported as the SAE. For example, if a subject is hospitalized to undergo coronary bypass surgery, record the heart condition that necessitated the bypass as the SAE.

Hospitalizations for the following reasons do not require reporting:
- for diagnostic or elective surgical procedures for preexisting conditions
- required to allow efficacy measurement for the study
- for scheduled therapy of the target disease of the study.

e. Pregnancy

If a female subject becomes pregnant while receiving investigational therapy or within 30 days of awareness after the last dose of study drug, a report should be completed and expeditiously submitted to R-Pharm US, Inc.. Follow-up by the Principal Investigator/Sponsor to obtain the outcome of the pregnancy should also occur. Abortion, whether accidental, therapeutic, or spontaneous, should always be classified as serious, and expeditiously reported as an SAE. Similarly, any congenital anomaly/birth defect in a child born to a female subject exposed to ixabepilone, bevacizumab, or the combination should be reported as an SAE as outlined in section 12.0.

f. Post-Study Adverse Events

The Principal Investigator/Sponsor and Sub-Site Principal Investigators should expeditiously report any SAE occurring after a subject has completed or discontinued study participation if attributed to prior exposure to ixabepilone, bevacizumab, or the combination. If the investigator should become aware of the development of cancer or a congenital anomaly in a subsequently conceived offspring of a female subject who participated in the study, this should be reported as an SAE.

g. Reconciliation

The Principal Investigator/Sponsor is responsible to monitor successful transmission of SAE reports to R-Pharm US, Inc. and the sponsor will conduct reconciliation on an as-needed basis to make sure that all SAEs are received and captured in the R-Pharm US, Inc. safety database.

11.11 Study Close-Out Reports

Any Clinical Study Report (final study report), abstracts or literary articles that are a result of the study should be sent to R-Pharm US, Inc. for review.

12.0 PROCEDURES FOR REPORTING UNANTICIPATED PROBLEMS INVOLVING RISKS TO SUBJECTS OR OTHERS, INCLUDING ADVERSE EVENTS (UPIRSOs)

12.1 Expedited Reporting of UPISROs Occurring at Yale

AEs classified as “serious” and “unexpected” that are possibly, probably, or definitely attributed to drug administration, or SAEs whose frequency exceeds expectations, require expeditious handling and reporting.

Serious Adverse Event (SAE)
Any adverse event that results in any of the following outcomes:

- death,
- a life-threatening experience,
- inpatient hospitalization or prolongation of existing hospitalization,
- a persistent or significant disability/incapacity,
- a congenital anomaly/birth defect, or
- any other adverse event that, based upon appropriate medical judgment, may jeopardize the subject's health and may require medical or surgical intervention to prevent one of the other outcomes listed in this definition.

The PI will promptly investigate all safety information related to an adverse experience. If the results of the PI's investigation show an adverse drug experience not initially determined to be reportable (based on whether the event is serious, unexpected, and associated with drug administration) is so reportable, the PI will report such experience. Follow-up information to a safety report shall be submitted as soon as the relevant information is available.

Reporting to the Yale Human Investigation Committee

Timeframe for Reporting

1. Events that may require a temporary or permanent interruption of study activities by the Principal Investigator/Sponsor to avoid potential harm to subjects should be reported to the IRB **immediately** (if possible), followed by a written report to the IRB using the UPIRSO Reporting Form (710 FR 4) **no more than 5 calendar days** after the Yale Principal Investigator becomes aware of the event**.**

2. Internal Events (defined above) should be reported to the IRB using the UPIRSO Reporting Form (710 FR 4) **within 5 calendar days** of the Principal Investigator/Sponsor becoming aware of the event.

3. External Events (defined above) should be reported to the IRB using the UPIRSO Reporting Form (710 FR 4) **within 15 calendar days** of the Principal Investigator/Sponsor becoming aware of the event ONLY IF either of the following are true:

   (a) The Principal Investigator/Sponsor has concluded that an immediate change to the protocol is necessary to address the risks raised by the event, and R-Pharm Us, Inc. agreed to the changes

   OR

   (b) A monitoring entity (e.g., an external IRB at the site where the problem or event occurred, the sponsor, or the Yale Cancer Center DSMB) has required modifications/amendments to the research protocol or consent documents as a result of the event.

For all reports of external events, the UPIRSO Reporting Form (710 FR 4) must include the following information:

(a) a clear explanation of why the event or series of events has been determined to meet criteria for reporting;

(b) a description of the proposed protocol changes and any corrective actions to be taken by the PI in response to the external event; and

(c) any aggregated data and an analysis or summary from the sponsor or DSMB, when applicable and available, sufficient to explain the significance of the event or series of events in order to ensure the information is interpretable and relevant to the IRB's task of protecting the rights and welfare of human participants.

Reporting to Investigators at Collaborating Sites

The Principal Investigator/Sponsor will notify all participating investigators in a written safety report of any adverse experience **associated with the use of the drug** that is both **serious** and **unexpected** as soon as possible and in no event later than 15 calendar days after the sponsor's (PI's) initial receipt of the information. [21CFR312.32(c)]

12.2 Sub-Site Principal Investigator SAE Reporting Requirements

The collaborating investigator (Sub-Site PI) in a multi-center trial will report **serious**, **unexpected** adverse events occurring at their site to the Principal Investigator/Sponsor **regardless of attribution** immediately (if possible) of knowledge of the event. Full written reports should be submitted to the Principal Investigator/Sponsor within 5 calendar days of initial knowledge of the report. The Principal Investigator/Sponsor will assess expectedness per FDA guidance below:

Per the Guidance for Industry and Investigators: Safety Reporting Requirements for INDs and BA/BE studies: The Principal Investigator/Sponsor is responsible for determining whether event meets the definition of "unexpected," based on whether the event is listed in the investigator brochure; or if an investigator brochure is not required or available, is not consistent with the risk information described elsewhere in the general investigational plan or elsewhere in the current application.

A Medwatch 3500A shall be used for reporting this event following the instructions outlined in section 12.3. This information will also need to be recorded in Oncore Clinical Trials Management System in the SAE section. Information entered into Oncore should directly reflect the Medwatch 3500A submission, and, additionally, will capture AE duration (i.e., start and stop dates), severity, outcome, regulatory seriousness criteria, treatment required, and action taken.

Send SAEs to the Principal Investigator/Sponsor:

Name: Alessandro Santin, MD
Phone: 203-737-4450

Email: alessandro.santin@yale.edu
Fax: 203-737-4339

12.3 SAE reporting to R-Pharm US, Inc.

The Principal Investigator/Sponsor shall report all SAEs and non-serious AEs which are relevant to a reported SAE by email using an SAE form as detailed below in accordance with the following timelines:

- within five (5) calendar days upon receipt by the Principal Investigator/Sponsor of initial and follow-up SAEs containing at least one fatal or immediately life-threatening event;
- within ten (10) calendar days upon receipt of any other initial and follow-up SAEs.

R-Pharm US, Inc.
Phone: 844.586.8953
Email: DrugSafety@propharmagroup.com

For each adverse event, the Sub-Site Principal Investigator will provide the onset date, end date, graded severity, treatment required, outcome, seriousness, and action taken with the investigational drug, with oversight by the Principal Investigator/Sponsor. The Principal Investigator/Sponsor will determine the relationship to ixabepilone, bevacizumab, or the combination and expectedness with the investigational drug to all AEs as defined in the listed adverse event section of the product's package inserts.

The investigator does not need to actively monitor patients for adverse events once the clinical trial has ended. However, if the investigator becomes aware of an SAE(s) that occurred after the patient has completed the clinical trial (including any protocol specified follow-up period), it should be reported to R-Pharm US, Inc., if the investigator considers it as relevant to the study drug.

MedWatch 3500A Reporting Guidelines:
In addition to completing appropriate patient demographic and suspect medication information, the report should include the following information within the Event Description (section B5 or G8) of the MedWatch 3500A form:

- Protocol description (and number, if assigned)
- Description of event, severity, treatment, outcome, start and stop dates, regulatory seriousness criteria, and action taken,
- Supportive laboratory results and diagnostics
- Investigator's assessment of the relationship of the adverse event to each investigational product and suspect medication

Follow-up information:
Additional information may be added to a previously submitted report by any of the following methods:

- Adding to the original MedWatch 3500A report and submitting it as follow-up
- Adding supplemental summary information and submitting it as follow-up with the original MedWatch 3500A form
- Summarizing new information and faxing it with a cover letter including patient identifiers (i.e. D.O.B. initial, patient number), protocol description and number, if assigned, brief adverse event description, and notation that additional or follow-up information is being submitted (The patient identifiers are important so that the new information is added to the correct initial report)

Occasionally the study sponsor R-Pharm US, Inc. may contact the reporter for additional information, clarification, or current status of the patient for whom an adverse event was reported. For questions regarding SAE reporting, you may contact the R-Pharm US, Inc. Safety representative. Relevant follow-up information should be submitted to R-Pharm US, Inc. as soon as it becomes available and/or upon request.

MedWatch 3500A (Mandatory Reporting) form is available at http://www.fda.gov/medwatch/getforms.html

At all times, the Remote Monitoring Coordinator is available to facilitate submissions and answer any questions regarding the process for all sub site staff.

13.0 MULTI-SITE MANAGEMENT AND COORDINATION

13.1 Overview

This is a multi-site trial where **Dr. Alessandro D. Santin** is the lead Principal Investigator/Sponsor for Yale University and for all non-Yale sites. The Gynecological Oncology research team at Yale University consisting of a Multi-site Research Coordinator, Remote Monitoring Coordinator, Multi-site Data Manager, Clinical Research Nursing Coordinator, Specimen Procurement Coordinator and a Multi-site Administrative Assistant will provide multicenter research support for all sites involved in this project. The YCCI clinical trials management support staff will provide clinical database systems support and training through the use of Oncore Clinical Trials Management System.

13.2 Initiation of Study

Once sub-site IRB approval has been obtained and all required start up documents have been submitted, Dr. Alessandro D. Santin will perform a Site Initiation Visit (SIV) prior to enrollment of study subjects. This will be either on-site or via teleconference with each participating site PI and staff available. Members of the Gynecologic Oncology research team will be available to answer all protocol questions. Once this has taken place with the participating site they may enroll subjects into the protocol. All pre and post SIV documents will be collected and stored in accordance with the Gynecologic Oncology Research SOP for collection of regulatory documents.

13.2.1 Investigational Site Training

Dr. Alessandro D. Santin or an appointed designee will provide investigational staff training prior to study initiation. Training topics will include but are not limited to: Good Clinical Practice (GCP), AE reporting, study details and procedure, study documentation, specimen procurement and shipping, informed consent, and enrollment. Sub sites are supplied with telephone and e-mail contact for all personnel and encouraged to contact with any questions regarding the conduct of the protocol.

13.2.2 Data Collection

Sites will use Oncore Clinical Trials Management System for all data associated with the protocol. Data managers at participating centers will receive Oncore access once they are designated by the site PI, complete an Oncore access request and submit HIPAA training certification. Individuals will receive Oncore training by a member of the YCCI clinical trials management support staff. Subjects entered into the system will be identified by a subject number assigned after randomization. The confidentiality of records that could identify subjects will be protected, respecting the privacy and confidentiality rules in accordance with the applicable regulatory requirement(s). The database will be monitored by the Multi-site Data manager and the Remote-site Monitoring Coordinator to ensure that data is entered in a timely manner.

13.3 Monitoring

Dr. Alessandro D. Santin or an appointed designee must be allowed to visit all study site locations periodically to assess the data quality and study integrity. On-site they will review study records and directly compare them with source documents, discuss the conduct of the study with the investigator, and verify that the facilities remain compliant.

In addition, remote monitoring of data may be performed periodically requiring the site to submit de-identified patient data for comparison to the Remote-site Monitoring Coordinator. The study may be audited by the Yale University DSMB internal auditors. Yale University DSMB audit reports will be kept confidential.

13.4 Study Enrollment Procedures

Any modification of the original consent document provided to participating sites by the Coordinating Center must be approved in advance by the Coordinating Center. Rationale will be provided with any request for a change. A copy of each site's IRB and Coordinating Center approved informed consent document must be on file at the Gynecological Oncology Regulatory office before subjects may be enrolled.

To register eligible patients on this study, each site will contact the Multi-site Coordinator; Lisa Baker, RN (203-785-6398) or Martha Luther, RN (203-737-2781) and provide the signed and dated eligibility checklist, completed signature page of the consent form and additional source documents if requested by the Principal Investigator or Multi-site Coordinator. Once the Principal Investigator or Multi-site Coordinator verifies eligibility, a unique subject study number will be issued. The subject will not be identified by name.

13.5 Responsibilities of the Lead Investigator Dr. Alessandro D. Santin:

The lead investigator is responsible for the overall conduct of the study at all participating sites and for monitoring the safety and quality of the data as well as compliance to the protocol and with applicable federal regulations and Good Clinical Practice (GCP).

The lead investigator will monitor accrual rates at all sites for adequate progress.
The Lead Investigator will ensure appropriate coordination, submission and approval of the protocol as well as the consent documents and any subsequent amendments at all sites. There will be only one version of these documents that will be used by all participating institutions and the lead investigator is responsible for assuring that the correct versions are used by all participating institutions.

No additional sites will be added to the study without a proposed amendment and review and approval by the Yale Cancer Center Protocol Review Committee and the Yale IRB (HIC).

The lead investigator has the authority to suspend accrual at any site not complying with this protocol, including not submitting data in a timely manner. Any suspension of accrual will be reported to the Yale University Data Safety Monitoring Committee (DSMC) as well as to the Yale University HHRP. Site principal investigators must report the suspension to their IRB.

13.6 Responsibilities of the Coordinating Center

The coordinating center, under the direction of the lead investigator Dr. Alessandro D. Santin, MD, is responsible to ensure that each participating site has the appropriate assurance on file with the Office for Human Research Protection (OHRP) or their local/central Institutional Review Board (IRB). The coordinating center is responsible for obtaining copies of OHRP/IRB assurance for each site prior to enrollment of subjects at the site.

13.7 IRB Approvals

The Coordinating Center is responsible to ensure that no patients are entered on study without full IRB approval and that IRB re-approval is appropriately maintained. A copy of the IRB approval document from each participating institution will be obtained by the Coordinating Center prior to activation of the study at any site. Documentation of reapproval must be provided to the Coordinating Center in a timely manner or registration will be halted at any site in which a current continuing approval is not on file at the Coordinating Center.

13.8 Amendments and Consents

The Coordinating Center will maintain a copy of all amendments, consent forms, and approvals from each site. Consent forms will be reviewed and approved by the Coordinating Center to ensure consistency with the Yale IRB approved consent. Should changes to the protocol or consent become necessary, protocol amendments will be issued by the Coordinating Center to all sites for local site approval prior to implementation, unless there is an apparent immediate hazard to a subject or the subject’s best interest is endangered. Any such deviation from the approved protocol will be promptly reported to the lead investigator.

13.9 Responsibilities of Participating Sites:

The principal investigator at each site is responsible for the overall conduct of the study at their site and for monitoring the safety and quality of the data as well as compliance to the protocol and with applicable federal regulations and (GCP).

The principal investigator at each site is responsible for assuring that all the required data is collected and entered onto the e-CRFs using Oncore Clinical Trials Management Systems in accordance with study-specific requirements and that the data submission and reporting timelines are met. The coordinating center will perform onsite monitoring periodically to ensure adherence to the protocol as well as regulatory compliance.

14.0 STATISTICAL CONSIDERATIONS

Study Design and Primary Objective: This is a randomized, two-arm, open-label Phase II multicenter study. Its design reflects its overarching goal: to examine the effects of adding bevacizumab to ixabepilone for the treatment of patients who have recurrent or persistent platinum-resistant/refractory epithelial (non-mucinous) ovarian, fallopian tube, or primary peritoneal cancer. Its primary objective is to assess whether adding bevacizumab to ixabepilone improves progression-free survival in its target population. Study participants will be stratified by (a) study site and (b) previous receipt of bevacizumab prior to randomization. Up to 88 participants will be randomly assigned at a 1:1 allocation ratio to the control arm (ixabepilone alone) or experimental arm (ixabepilone +bevacizumab) using a dynamic randomization procedure that minimizes stratification-factor imbalance between the treatment arms. Patient registration will be accomplished in the usual fashion.

Secondary objectives:
- To compare the experimental arm to the control arm for increases in objective response rate (ORR) and durable disease control rate (DDCR).
- To compare the experimental arm to the control arm for an increase in overall survival (OS)
- To assess the safety profile of ixabepilone with bevacizumab in patients with recurrent or persistent platinum-resistant/refractory epithelial (non-mucinous) ovarian, fallopian tube, or primary peritoneal cancer.

Exploratory/correlative objectives:
- To characterize number, length and composition (e.g., class III β-tubulin expression) of microtentacles (McTNs) isolated from circulating tumor cells isolated from whole blood of patients undergoing treatment with ixabepilone with or without bevacizumab, and correlate with best response, PFS, and OS.
- To observe McTNs on circulating tumor cells in blood using a novel polyelectrolyte multi-layer (PEM) tethering technology.
- To correlate *ex vivo* response of McTNs to drug treatment with clinical response in order to develop a real-time assay to predict response to therapy.
- To explore use of circulating tumor (ct) DNA as a biomarker for disease response and compare its performance to CA-125.

- To examine whether clinical response to ixabepilone with or without bevacizumab differs between high and low expressors of class III β-tubulin.

14.1 Sample-Size Justification and Accrual Goals

The primary objective of the study is to compare the experimental arm to the control arm for evidence of an increase in progression-free survival (PFS). We estimate that median PFS on the control arm will be approximately 5 months. This estimate is based on the observation of a 4.4-month median PFS in GOG 126M, a phase II study of ixabepilone monotherapy in platinum-resistant ovarian and primary peritoneal cancers.[37] In addition, and based on our own observational data with the addition of bevacizumab to ixabepilone[42] along with results from the AURELIA trial,[9] we propose that the addition of bevacizumab to ixabepilone will lead to a 2-fold increase (to 10 months) in median PFS (or 50% reduction in hazard of PFS events). Treatment arms will be compared for the proposed PFS improvement via one-sided log-rank test. We require this test to have at least 80% power at 5% alpha to detect the 2-fold increase while allowing for a single interim analysis for efficacy and futility. Calculations conducted in East version 6.4 software (Cytel, Inc, Cambridge, MA, USA) using the null-variance estimator along with O'Brien-Fleming spending functions for both alpha and beta indicate that 28 PFS events in the interim analysis and 56 PFS events in the final analysis will provide the one-sided log-rank test with 80.8% overall power at 4.7% overall alpha, thereby meeting our requirements.

To achieve the required 56 PFS events in a timely manner while allowing for up to 10% dropouts among recruited subjects, we plan to recruit subjects for 17.6 months at a target accrual rate of 5 subjects/month to reach a final recruitment goal of 88 subjects before dropout (80 subjects after dropout). If our target accrual rate is met from the beginning, and if the control arm has the expected median PFS of 5 months, then the 56th PFS event is projected to occur no later than 21.8 months after the study opens. The study's total duration is thus projected to have a maximum of 21.8 months.

14.2 Analysis Plan in support of the Primary Objective

Progression-free survival (PFS), the primary endpoint, will be defined as the length of time from randomization to disease recurrence, disease progression, or death for any reason. Subjects who are alive and who did not experience disease recurrence or progression by the end of the study will be censored for PFS at the date of their last contact. The experimental arm will be compared to the control arm for improvement in PFS via one-sided log-rank test. This test will be conducted once for the interim analysis and once for the final analysis.

14.2.1 *Interim Analysis.* A formal interim analysis for efficacy and futility will take place when 28 PFS events have occurred among enrolled study subjects. Based on previously stated assumptions (section 14.1), we would project that the 28th PFS event will occur sometime between when the 58th and 67th subject has enrolled, or between 11.5 and 13.2 months after the study opens. Unless there are toxicity concerns or extenuating circumstances, the study will remain open during this interim analysis. To conduct the interim analysis, the experimental arm will be compared to the control arm via one-sided log-rank test.

- If the test yields a Z-score equal to or more negative than –2.538 (equivalent to a one-sided *P*≤0.0056 and hazard ratio ≤0.383), then the experimental arm (ixabepilone + bevacizumab) will be declared promising enough to warrant closing the study early for efficacy.
- On the other hand, if the test yields a Z-score equal to or more positive than –0.3204 (equivalent to a one-sided *P*≥0.3743 and hazard ratio ≥0.886), then that result will be considered negative enough to warrant closing the study early for futility.

Findings of either kind that warrant closing the study early will not be binding, but will be evaluated for closure in conjunction with safety, toxicity, and other relevant evidence.

14.2.2 *Final Analysis.* If the study remains open after the interim analysis, then the final analysis in support of the primary objective will take place when 56 PFS events have occurred. To conduct the final analysis, the experimental arm will be compared to the control arm via one-sided log-rank test.

- If the test yields a Z-score equal to or more negative than –1.6621 (equivalent to a one-sided *P*≤0.0482 and hazard ratio ≤0.641), then this study will be declared a success, and the combination of ixabepilone + bevacizumab will be considered worthy of further study in a Phase III trial.

The above final analysis will be accompanied by Kaplan-Meier estimates of PFS medians and quartiles on each arm. It also will be followed up with re-analysis using a stratified version of the one-sided log-rank test that has study site and receipt of prior bevacizumab as stratification factors.

14.3 Analysis Plan in support of the Secondary and Exploratory Objectives

Secondary objectives.

- ORR and DDCR will each be summarized by treatment arm as number and proportion of occurrences, then compared for increase with bevacizumab via one-sided chi-square test at alpha=0.05.
- OS will be plotted as Kaplan-Meier curves, summarized by treatment arm as medians and quartiles, then compared for increase with bevacizumab via one-sided log-rank test at alpha=0.05. Both the unstratified and stratified versions of the log-rank test will be used. The stratified version will have study site and prior bevacizumab status as the stratification factors.

Exploratory objectives. See also Section 5.2, Exploratory Objectives.

- Both PFS and OS will be analyzed via multivariate Cox regression with treatment arm and additional predictor variables consisting of class III β-tubulin status, baseline levels of CA-125 and *TP53*-mutated ct-DNA, and baseline values of McTN characteristics such as number, length, and composition. These analyses will assess each additional predictor for its potential as a prognostic biomarker.
- Both ORR and DDCR will be analyzed via multivariate logistic regression with treatment arm and additional predictor variables consisting of class III β-tubulin status, baseline levels of CA-125 and *TP53*-mutated ct-DNA, and baseline values of McTN characteristics

such as number, length, and composition. These analyses will assess each additional predictor for its potential as a prognostic biomarker of response to treatment.

- Results of the proposed McTN chemosensitivity assay (Section 5.2.2), measured at baseline, will be used as predictor variables in logistic regression (with ORR and DDCR as outcomes) to assess how strong the correlation is between patients' tumor-cell response to drugs and their clinical response to treatment.
- The behavior during treatment of longitudinally measured McTN characteristics, *TP53*-mutated ct-DNA, and CA-125 will be studied using trajectory plots in conjunction with longitudinal-analysis approaches such as mixed-models analysis, logistic regression with generalized estimating equations, and other procedures as suggested by the nature of the data. The same longitudinally measured markers may also be entered as time-dependent covariates into Cox-regression models to study how strongly their behavior during treatment is associated with PFS and OS. These analyses will be an important component of evaluating how effective the biomarkers may be at signaling and/or presaging changes in the patient's health in a patient-monitoring situation.

14.4 Evaluability for Efficacy and Toxicity:

Only those patients who are deemed "ineligible" or who receive no therapy will be eliminated from the analysis. All patients who receive any therapy will be evaluated for both treatment efficacy and toxicity. While on occasion, circumstances may prevent the determination of treatment efficacy, such patients will be included in the analysis and labeled as "unknown". This category will be listed and be reflected in the calculation of the response rate.

15.0 BIBLIOGRAPHY

1. Siegel, R. L., Miller, K. D. & Jemal, A. Cancer statistics, 2016. *CA. Cancer J. Clin.* **66,** 7–30 (2016).
2. Kosary, C. L. in *In: Ries LAG, Young JL, Keel GE, et al (eds). SEER Survival Monograph* 133–144
3. Ozols, R. F. *et al.* Phase III trial of carboplatin and paclitaxel compared with cisplatin and paclitaxel in patients with optimally resected stage III ovarian cancer: a Gynecologic Oncology Group study. *J. Clin. Oncol. Off. J. Am. Soc. Clin. Oncol.* **21,** 3194–3200 (2003).
4. du Bois, A. *et al.* A randomized clinical trial of cisplatin/paclitaxel versus carboplatin/paclitaxel as first-line treatment of ovarian cancer. *J. Natl. Cancer Inst.* **95,** 1320–1329 (2003).
5. Cannistra, S. A. Cancer of the Ovary. *N. Engl. J. Med.* **329,** 1550–1559 (1993).
6. Thigpen, J. T., Blessing, J. A., Ball, H., Hummel, S. J. & Barrett, R. J. Phase II trial of paclitaxel in patients with progressive ovarian carcinoma after platinum-based chemotherapy: a Gynecologic Oncology Group study. *J. Clin. Oncol. Off. J. Am. Soc. Clin. Oncol.* **12,** 1748–1753 (1994).
7. Markman, M. *et al.* Second-line platinum therapy in patients with ovarian cancer previously treated with cisplatin. *J. Clin. Oncol. Off. J. Am. Soc. Clin. Oncol.* **9,** 389–393 (1991).
8. Markman, M. & Bookman, M. A. Second-line treatment of ovarian cancer. *The Oncologist* **5,** 26–35 (2000).
9. Pujade-Lauraine, E. *et al.* Bevacizumab combined with chemotherapy for platinum-resistant recurrent ovarian cancer: The AURELIA open-label randomized phase III trial. *J. Clin. Oncol. Off. J. Am. Soc. Clin. Oncol.* **32,** 1302–1308 (2014).
10. Jain, R. K. Normalization of tumor vasculature: an emerging concept in antiangiogenic therapy. *Science* **307,** 58–62 (2005).
11. Poveda, A., Selle, F. & Hilpert, F. Weekly paclitaxel, pegylated liposomal doxorubicin, or topotecan +/- bevacizumab in platinum-resistant ovarian cancer: analysis by chemotherapy cohort in the GCIG-AURELIA randomised phase III trial [abstract LBA26]. (2012).
12. Burger, R. A., Sill, M. W., Monk, B. J., Greer, B. E. & Sorosky, J. I. Phase II trial of bevacizumab in persistent or recurrent epithelial ovarian cancer or primary peritoneal cancer: a Gynecologic Oncology Group Study. *J. Clin. Oncol. Off. J. Am. Soc. Clin. Oncol.* **25,** 5165–5171 (2007).

13. Burger R.A., Brady M.F., Bookman M.A., Fleming G.F., Monk B.J., et al. Gynecologic Oncology Group Incorporation of bevacizumab in the primary treatment of ovarian cancer, a GOG study. N. Engl. J. Med. **365,** 2473-2483 (2011).

14. Perren T.J., Swart A.M., Pfisterer J., Ledermann J.A., Pujade-Lauraine E., et al. A phase 3 trial of bevacizumab in ovarian cancer. *.N Eng.l J. Med*. **365(26)**, 2484–2496 (2011).

15. Backes F.J., Richardson D.L., McCann G.A., Smith B., Salani R., et al. Should bevacizumab be continued after progression on bevacizumab in recurrent ovarian cancer*? Int. J. Gynecol. Cancer* **23(5)**, 833-838 (2013).

16. Bennouna J., Sastre J., Arnold D., Österlund P., Greil R., et al. ML18147 Study Investigators. Continuation of bevacizumab after first progression in metastatic colorectal cancer (ML18147): a randomised phase 3 trial. *Lancet Oncol*. **14(1)**, 29–37 (2013).

17. National Institutes of Health. Bevacizumab Beyond Progression in Platinum Sensitive Ovarian Cancer (MITO16MANGO2b).In ClinicalTrials.gov [Internet]. Bethesda (MD): National Library of Medicine (US). 2000- [cited 2002 Feb 27]. Available from: http://clinicaltrials.gov/show/NCT01802749 NLM Identifier: NCT01802749.

18. McClung E.C., Wenham R.M. Profile of bevacizumab in the treatment of platinum-resistant ovarian cancer: current perspectives. *Int. J. Womens Health*. **8**, 59-75 (2016).

19. Caplow, M. & Zeeberg, B. Dynamic properties of microtubules at steady state in the presence of taxol. *Eur. J. Biochem. FEBS* **127,** 319–324 (1982).

20. English, D. P., Roque, D. M. & Santin, A. D. Class III b-tubulin overexpression in gynecologic tumors: implications for the choice of microtubule targeted agents? *Expert Rev. Anticancer Ther.* **13,** 63–74 (2013).

21. Parker, A. L., Kavallaris, M. & McCarroll, J. A. Microtubules and their role in cellular stress in cancer. *Front. Oncol.* **4,** 153 (2014).

22. Janke, C. & Bulinski, J. C. Post-translational regulation of the microtubule cytoskeleton: mechanisms and functions. *Nat. Rev. Mol. Cell Biol.* **12,** 773–786 (2011).

23. Kavallaris, M. Microtubules and resistance to tubulin-binding agents. *Nat. Rev. Cancer* **10,** 194–204 (2010).

24. McCourt, C. *et al.* Is there a taxane-free interval that predicts response to taxanes as a later-line treatment of recurrent ovarian or primary peritoneal cancer? *Int. J. Gynecol. Cancer Off. J. Int. Gynecol. Cancer Soc.* **19,** 343–347 (2009).

25. Orr, G. A., Verdier-Pinard, P., McDaid, H. & Horwitz, S. B. Mechanisms of Taxol resistance related to microtubules. *Oncogene* **22,** 7280–7295 (2003).

26. Gottesman, M. M. Mechanisms of cancer drug resistance. *Annu. Rev. Med.* **53,** 615–627 (2002).

27. Blagosklonny, M. V. & Fojo, T. Molecular effects of paclitaxel: myths and reality (a critical review). *Int. J. Cancer* **83,** 151–156 (1999).

28. Dumontet, C. & Sikic, B. I. Mechanisms of action of and resistance to antitubulin agents: microtubule dynamics, drug transport, and cell death. *J. Clin. Oncol. Off. J. Am. Soc. Clin. Oncol.* **17,** 1061–1070 (1999).

29. Drukman, S. & Kavallaris, M. Microtubule alterations and resistance to tubulin-binding agents (review). *Int. J. Oncol.* **21,** 621–628 (2002).

30. Roque, D. M. *et al.* Tubulin-β-III overexpression by uterine serous carcinomas is a marker for poor overall survival after platinum/taxane chemotherapy and sensitivity to epothilones. *Cancer* **119,** 2582–2592 (2013).

31. Roque, D. M. *et al.* Class III β-tubulin overexpression in ovarian clear cell and serous carcinoma as a maker for poor overall survival after platinum/taxane chemotherapy and sensitivity to patupilone. *Am. J. Obstet. Gynecol.* **209,** 62.e1-9 (2013).

32. Roque, D. M. *et al.* Class III β-tubulin overexpression within the tumor microenvironment is a prognostic biomarker for poor overall survival in ovarian cancer patients treated with neoadjuvant carboplatin/paclitaxel. *Clin. Exp. Metastasis* **31,** 101–110 (2014).

33. Carrara, L. *et al.* Differential in vitro sensitivity to patupilone versus paclitaxel in uterine and ovarian carcinosarcoma cell lines is linked to tubulin-beta-III expression. *Gynecol. Oncol.* **125,** 231–236 (2012).

34. Akbari, V., Moghim, S. & Reza Mofid, M. Comparison of Epothilone and Taxol Binding in Yeast Tubulin using Molecular Modeling. *Avicenna J. Med. Biotechnol.* **3,** 167–175 (2011).

35. Lee, F. Y. F. *et al.* Preclinical discovery of ixabepilone, a highly active antineoplastic agent. *Cancer Chemother. Pharmacol.* **63,** 157–166 (2008).

36. FDA Approval for Ixabepilone. *National Cancer Institute* Available at: https://www.cancer.gov/about-cancer/treatment/drugs/fda-ixabepilone. (Accessed: 4th October 2016)

37. De Geest, K. *et al.* Phase II clinical trial of ixabepilone in patients with recurrent or persistent platinum- and taxane-resistant ovarian or primary peritoneal cancer: a gynecologic oncology group study. *J. Clin. Oncol. Off. J. Am. Soc. Clin. Oncol.* **28,** 149–153 (2010).

38. Awada, A. *et al.* Phase I dose escalation study of weekly ixabepilone, an epothilone analog, in patients with advanced solid tumors who have failed standard therapy. *Cancer Chemother. Pharmacol.* **63,** 417–425 (2009).

39. Ferrandina, G. *et al.* Class III beta-tubulin overexpression is a marker of poor clinical outcome in advanced ovarian cancer patients. *Clin. Cancer Res. Off. J. Am. Assoc. Cancer Res.* **12,** 2774–2779 (2006).

40. Lee, F. Y. F. *et al.* Synergistic antitumor activity of ixabepilone (BMS-247550) plus bevacizumab in multiple in vivo tumor models. *Clin. Cancer Res. Off. J. Am. Assoc. Cancer Res.* **14,** 8123–8131 (2008).

41. Yamaguchi, H., Chen, J., Bhalla, K. & Wang, H.-G. Regulation of Bax activation and apoptotic response to microtubule-damaging agents by p53 transcription-dependent and -independent pathways. *J. Biol. Chem.* **279,** 39431–39437 (2004).

42. Roque, D. M. *et al.* Weekly ixabepilone with or without biweekly bevacizumab in the treatment of recurrent or persistent uterine and ovarian/primary peritoneal/fallopian tube cancers: A retrospective review. *Gynecol. Oncol.* **137,** 392–400 (2015).

43. Whipple, R. A. *et al.* Vimentin filaments support extension of tubulin-based microtentacles in detached breast tumor cells. *Cancer Res.* **68,** 5678–5688 (2008).

44. Whipple, R. A., Cheung, A. M. & Martin, S. S. Detyrosinated microtubule protrusions in suspended mammary epithelial cells promote reattachment. *Exp. Cell Res.* **313,** 1326–1336 (2007).

45. Balzer, E. M., Whipple, R. A., Cho, E. H., Matrone, M. A. & Martin, S. S. Antimitotic chemotherapeutics promote adhesive responses in detached and circulating tumor cells. *Breast Cancer Res. Treat.* **121,** 65–78 (2010).

46. Matrone, M. A., Whipple, R. A., Balzer, E. M. & Martin, S. S. Microtentacles tip the balance of cytoskeletal forces in circulating tumor cells. *Cancer Res.* **70,** 7737–7741 (2010).

47. Chakrabarti, K. R. *et al.* Lipid tethering of breast tumor cells enables real-time imaging of free-floating cell dynamics and drug response. *Oncotarget* **7,** 10486–10497 (2016).

48. FDA. Substantial equivalence determination for CA-125. Available at http://www.accessdata.fda.gov/cdrh_docs/reviews/K042731.pdf.

49. Bast, R. C. *et al.* A radioimmunoassay using a monoclonal antibody to monitor the course of epithelial ovarian cancer. *N. Engl. J. Med.* **309,** 883–887 (1983).

50. Myers, E. R. *et al.* Management of adnexal mass. *Evid. ReportTechnology Assess.* 1–145 (2006).

51. Jacobs, I. & Bast, R. C. The CA 125 tumour-associated antigen: a review of the literature. *Hum. Reprod. Oxf. Engl.* **4,** 1–12 (1989).

52. Bast, R. C. CA 125 and the detection of recurrent ovarian cancer: a reasonably accurate biomarker for a difficult disease. *Cancer* **116,** 2850–2853 (2010).

53. Martignetti, J. A. *et al.* Personalized ovarian cancer disease surveillance and detection of candidate therapeutic drug target in circulating tumor DNA. *Neoplasia N. Y. N* **16,** 97–103 (2014).

54. Schwarzenbach, H., Hoon, D. S. B. & Pantel, K. Cell-free nucleic acids as biomarkers in cancer patients. *Nat. Rev. Cancer* **11,** 426–437 (2011).

55. Dawson, S.-J. *et al.* Analysis of circulating tumor DNA to monitor metastatic breast cancer. *N. Engl. J. Med.* **368,** 1199–1209 (2013).

56. Freidin, M. B. *et al.* Circulating tumor DNA outperforms circulating tumor cells for KRAS mutation detection in thoracic malignancies. *Clin. Chem.* **61,** 1299–1304 (2015).

57. Reinert, T. *et al.* Analysis of circulating tumour DNA to monitor disease burden following colorectal cancer surgery. *Gut* **65,** 625–634 (2016).

58. Hamakawa, T. *et al.* Monitoring gastric cancer progression with circulating tumour DNA. *Br. J. Cancer* **112,** 352–356 (2015).

59. Pereira, E. *et al.* Personalized Circulating Tumor DNA Biomarkers Dynamically Predict Treatment Response and Survival In Gynecologic Cancers. *PloS One* **10,** e0145754 (2015).

60. RainDrop™ Digital PCR System by RainDance Technologies, Inc. Available at: http://www.selectscience.net/products/raindrop%E2%84%A2-digital-pcr-system/?prodID=116161#tab-2. (Accessed: 5th October 2016)

61. Cancer Genome Atlas Research Network. Integrated genomic analyses of ovarian carcinoma. *Nature* **474,** 609–615 (2011).

62. Eisenhauer, E. A. *et al.* New response evaluation criteria in solid tumours: revised RECIST guideline (version 1.1). *Eur. J. Cancer Oxf. Engl. 1990* **45,** 228–247 (2009).

63. Morris, J. C. The Clinical Dementia Rating (CDR): current version and scoring rules. *Neurology* **43,** 2412–2414 (1993).

64. Soslow, R. A. Histologic subtypes of ovarian carcinoma: an overview. *Int. J. Gynecol. Pathol. Off. J. Int. Soc. Gynecol. Pathol.* **27,** 161–174 (2008).

65. McGuire, V., Jesser, C. A. & Whittemore, A. S. Survival among U.S. women with invasive epithelial ovarian cancer. *Gynecol. Oncol.* **84,** 399–403 (2002).

66. Hess, V. *et al.* Mucinous epithelial ovarian cancer: a separate entity requiring specific treatment. *J. Clin. Oncol. Off. J. Am. Soc. Clin. Oncol.* **22,** 1040–1044 (2004).

67. Mozzetti, S. *et al.* Class III beta-tubulin overexpression is a prominent mechanism of paclitaxel resistance in ovarian cancer patients. *Clin. Cancer Res. Off. J. Am. Assoc. Cancer Res.* **11,** 298–305 (2005).

68. Smith J.W. 2nd, Vukelja S., Rabe A., Wentworth-Hartung N., Koutrelakos N., et al. Phase II randomized trial of weekly and every-3-week ixabepilone in metastatic breast cancer patients. *Breast Cancer Res. Treat*. **142(2)**, 381-388 (2013).

69. Maitland, M. L. *et al.* Initial assessment, surveillance, and management of blood pressure in patients receiving vascular endothelial growth factor signaling pathway inhibitors. *J. Natl. Cancer Inst.* **102,** 596–604 (2010).

70. Common Terminology Criteria for Adverse Events (CTCAE) - CTCAE_4.03_2010-06-14_QuickReference_5x7.pdf. Available at: https://evs.nci.nih.gov/ftp1/CTCAE/CTCAE_4.03_2010-06-14_QuickReference_5x7.pdf. (Accessed: 3rd October 2016)

Appendix A:

FIGO Surgical Stages for Ovarian, Fallopian Tube, or Primary Peritoneal Cancer

IA Tumor limited to 1 ovary, capsule intact, no tumor on surface, negative washings.
IB Tumor involves both ovaries, otherwise like IA.
IC Tumor limited to 1 or both ovaries:
- IC1 Surgical spill
- IC2 Capsule rupture before surgery or tumor on ovarian, fallopian tube, or primary peritoneal surface
- IC3 Malignant cells in the ascites or peritoneal washings

IIA Extension and/or implant on uterus and/or fallopian tubes
IIB Extension to other pelvic intraperitoneal tissues

IIIA Positive retroperitoneal lymph nodes and/or microscopic metastasis beyond the pelvis
- IIIA1 Positive retroperitoneal lymph nodes only
  - IIIA1(i) Metastasis ≤ 10 mm
  - IIIA1(ii) Metastasis > 10 mm
- IIIA2 Microscopic, extrapelvic (above the brim) peritoneal involvement ± positive retroperitoneal lymph nodes

IIIB Macroscopic, extrapelvic, peritoneal metastasis ≤ 2 cm ± positive retroperitoneal lymph nodes.; includes extension to capsule of liver/spleen
IIIC Macroscopic, extrapelvic, peritoneal metastasis > 2 cm ± positive retroperitoneal lymph nodes; includes extension to capsule of liver/spleen

IVA Pleural effusion with positive cytology
IVB Hepatic and/or splenic parenchymal metastasis, metastasis to extraabdominal organs, including inguinal lymph nodes and lymph nodes outside of the abdominal cavity

Appendix B:
Performance Status Criteria

**ECOG Performance Status Scale**		**Karnofsky Performance Scale**	
Grade	Descriptions	Percent	Description
0	Normal activity. Fully active, able to carry on all pre-disease performance without restriction.	100	Normal, no complaints, no evidence of disease.
		90	Able to carry on normal activity; minor signs or symptoms of disease.
1	Symptoms, but ambulatory. Restricted in physically strenuous activity, but ambulatory and able to carry out work of a light or sedentary nature (e.g., light housework, office work).	80	Normal activity with effort; some signs or symptoms of disease.
		70	Cares for self, unable to carry on normal activity or to do active work.
2	In bed <50% of the time. Ambulatory and capable of all self-care, but unable to carry out any work activities. Up and about more than 50% of waking hours.	60	Requires occasional assistance, but is able to care for most of his/her needs.
		50	Requires considerable assistance and frequent medical care.
3	In bed >50% of the time. Capable of only limited self-care, confined to bed or chair more than 50% of waking hours.	40	Disabled, requires special care and assistance.
		30	Severely disabled, hospitalization indicated. Death not imminent.
4	100% bedridden. Completely disabled. Cannot carry on any self-care. Totally confined to bed or chair.	20	Very sick, hospitalization indicated. Death not imminent.
		10	Moribund, fatal processes progressing rapidly.
5	Dead.	0	Dead.

APPENDIX C

Shipping address for IHC slides and EDTA blood samples:

Alessandro D. Santin MD
Yale University School of Medicine
Department of Obstetrics, Gynecology & Reproductive Sciences
333 Cedar Street, LSOG Bld. Room 305, PO Box 208063
New Haven, CT, 06520-8063
Phone: 203-737-4450, Fax: 203-737-4339
E-mail: alessandro.santin@yale.edu

Shipping address for CellSave blood samples:

Jocelyn Reader, PhD
University of Maryland Greenebaum Cancer Center
University of Maryland School of Medicine
Division of Gynecologic Oncology, Dept. of Obstetrics/Gynecology & Reproductive Sciences
10th Floor, 655 W. Baltimore St.
Baltimore, MD 21201
Phone: 410-706-6247
E-mail: jreader@fpi.umaryland.edu

I. Summary of Specimen Requirements

1) An H&E stained slide of the tumor for Yale Pathology confirmatory assessment of histology
2) IHC-stained slide(s) for class III β-tubulin
3) If unable to supply (1) and (2) above, formalin-fixed, paraffin-embedded tumor tissue block OR 5 unstained slides
4) Whole blood specimens (20 mL in CellSave preservative tubes)
5) Whole blood specimens (10 mL in EDTA tube)

II. Time Points Deadlines and Recommendations

1) Please send the above at the time of patient enrollment in a FED-EX envelope for overnight delivery. A preprinted FED-EX label will be provided. In order to produce the label, a date of shipment is necessary. Please e-mail michele.montagna@yale.edu, martha.luther@yale.edu, and lisa.patriub@yale.edu with the date of shipment and a pre-printed label will be e-mailed back to you.
2) Whole blood (minimum 20 mL) drawn into CellSave preservative tubes (Janssen Diagnostics, Raritan, NJ) and 10 mL drawn in EDTA tubes for translational research studies will be collected before the first treatment (i.e., within 28 days of initiation of therapy), and 0-3 days before every other cycle. Blood samples should be shipped at room temperature to the addresses above the day the specimen is collected in a FED-EX envelope for overnight delivery. Circulating tumor cells are viable for only up to 96 hours at room temperature using this system. A preprinted FED-EX label will be provided. In order to produce the label, a date of shipment is necessary. Please e-mail michele.montagna@yale.edu, martha.luther@yale.edu and lisa.patriub@yale.edu with the date of shipment and a pre-printed label will be e-mailed back.
